# Psychosocial well-being in middle schoolers: effects of a school cycling program in the wake of COVID-19

**DOI:** 10.3389/fspor.2025.1646805

**Published:** 2025-11-07

**Authors:** Starla Murillo, Ashtyn Philipsheck, Fletcher Dementyev, Benjamin Bello-Sotto, Eshan Bhatt, Hunter Wilson, Kai Madison, Lauren Schuck, Seth A. Wiafe, Cian L. Brown, Esther J. Walker, Sean M. Wilson

**Affiliations:** 1 University of Redlands, Redlands, CA, United States; 2 Lawrence D Longo Center for Perinatal Biology, Loma Linda University School of Medicine, Loma Linda, CA, United States; 3 Outride, Morgan Hill, CA, United States; 4 Loma Linda University School of Public Health, Loma Linda, CA, United States; 5 Department of Educational Psychology, University of Oklahoma, Norman, OK, United States

**Keywords:** WHO-5, PSC-17-Y, adolescent mental health and well-being, school-based physical activity programmes, cycling intervention, post-pandemic well-being, modifiable lifestyle factors

## Abstract

**Introduction:**

The impact of the Riding for Focus (R4F) school-based cycling program and key risk factors on middle school students’ mental health was evaluated following COVID-19 lockdowns. Adolescents face growing mental health challenges that the R4F program aims to address by promoting physical activity and well-being.

**Methods:**

The study surveyed students from 31 U.S. schools, assessing mental health via the WHO-5 Well-Being Index and PSC-17-Y checklist. Non-parametric tests (Mann–Whitney U, Kruskal–Wallis ANOVA with Dunn's test) and effect size calculations (Cohen's d) were used; clinical risk assessments employed Fisher's Exact Test and Koopman scores with established cutoff values. Modifiable risk factors analyzed included physical activity, sleep, screen time, and breakfast habits. Linear regression evaluated dose-response relationships between these factors and wellness scores.

**Results:**

Participation in R4F was linked to a modest 5% boost in WHO-5 well-being scores; however, PSC-17-Y scores also increased slightly, contrary to previous findings, indicating more reported symptoms. Differences in outcomes were seen across gender and race/ethnicity. Notably, modifiable risk factors such as sleep, screen time, and physical activity showed clear dose-response relationships with mental health metrics.

**Discussion:**

Results suggest the R4F program may support adolescent mental health, though outcomes vary by demographic and lifestyle factors, highlighting a need for targeted, individualized interventions in youth populations.

## 1 Introduction

Mental health research, particularly focusing on adolescents, is of paramount importance to our society. Recent studies investigating the COVID-19 pandemic's impact on adolescent mental health and well-being are yielding new insights into the development of psychological disorders. Following the COVID-19 lockdowns, rates of general anxiety, depression, post-traumatic stress disorder, and social anxiety symptoms increased significantly (1–5) and have yet to return to pre-pandemic levels (6, 7). As of 2021, a staggering 42% of the youth population in the United States reported consistently feeling sad or hopeless, with nearly one-third experiencing poor mental health (8). Major depressive episodes and anxiety are among the most prevalent symptoms of poor mental health, with a substantial number of children and adolescents experiencing these conditions (9). A comprehensive review reveals that by age 14, approximately one-third of young teens already exhibit externalizing, internalizing, and attention behavioral issues, which rises to nearly half of all adolescents by age 18 (10).

Adolescent mental health and psychological well-being are influenced by multiple lifestyle, socioeconomic, and other factors, which interact to affect risk and outcomes. Key determinants include gender, race, socioeconomic status (SES), social interaction and engagement, screentime, sleep duration and physical activity (PA) levels (11, 12). Regular PA is closely linked to improved mental health and psychological well-being in children and adolescents (13–15). Research consistently shows that exercise enhances self-image, quality of life, and happiness, while also reducing psychological distress (16). Numerous studies support the role of exercise as both a preventive and therapeutic strategy for depression and anxiety (17, 18). The positive effects of PA are broad, ranging from the alleviation of negative emotions to neurobiological changes that support emotional regulation (19–21). Cycling as a form of exercise has been shown to promote a healthier physical well-being including, but not limited to, lower risks of a cardiovascular event (22–24), Type 2 Diabetes (25), and obesity (25).

Aerobic as well as resistance based PA, whether performed as a leisure activity or in school, is widely recognized as an effective method to combat poor mental, physical, and cognitive health in children, adolescents, and adults (13–16, 18, 19). Considering that adolescents in U.S. public schools spend an average of 180 days per year in school (26), well-designed school-based physical education (PE) programs present a valuable opportunity to help students meet the 1 h daily PA recommendations outlined in both U.S. Physical Activity Guidelines and WHO global standards (27, 28). Well-designed PE programs not only promote active lifestyle behaviors but also foster emotional intelligence, social skills, and long-term enthusiasm for PA (29, 30). However, these benefits are not always realized due to budget constraints, inconsistent program quality, or lack of accessibility (30–32). The COVID-19 pandemic further complicated the picture, as lockdowns and remote learning disrupted PA routines and contributed to declines in mental health, consistent with the established link between reduced PA and increased risk of mental health disorders (19, 33) Nevertheless, some forms of PA experienced rises in participation during the pandemic. Notably, cycling outdoors in the U.S. experienced a 16% increase between 2019 and 2020 (34), and generally saw an increase in prevalence in 2022 compared to 2020 (35). Unfortunately, participation rates among 10- to 17-year-olds continued to decline, with just under half reporting bicycling within the previous year. Young riders under 17 are most likely to use bicycles for recreation or a combination of recreation and transportation. However, engagement drops as adolescents approach driving age, largely due to increased academic pressures, participation in extracurricular activities, social influences, and persistent safety concerns (36). These and other barriers have contributed to a steady decline in biking frequency and participation over the last decade. For cycling to become a lifestyle choice rather than merely a transitional or recreational activity, systematic improvements including safer infrastructure, supportive social norms, and integration into daily routines are essential to keep riding accessible and appealing for adolescents and beyond (37, 38).

Cycling is particularly valuable because it supports physical endurance, bone and muscle health, mental well-being, and prevention of chronic diseases such as diabetes (39). Cycling has a number of distinctions that set it apart from other physical activities and sports, including that the bicycle can be used for recreation, transportation, as well as competition. Riding bicycles is low impact, which minimizes bone stress, it allows for participation across all ages and fitness levels, and cycling provides additional benefits of balance and lifelong accessibility relative to high impact and skill-based sports. Researchers support cycling as interventions that promote increased physical activity, which is crucial for youth health and combating sedentary lifestyles. School-based cycling programs have demonstrated improvements in moderate-to-vigorous physical activity, cycling skills, and physical fitness among children and adolescents (40–42). Learning to cycle through progressive skill building strategies, such as using balance bikes before learning to pedal, leads to more efficient cycling skill acquisition, reduced sedentary time, and improved leg strength and body composition (43, 44). Beyond physical benefits, cycling fosters prosocial development by providing opportunities for increased responsibility, confidence, leadership, and social connection (45, 46). Given the immense societal and economic burden of mental illness, which is estimated at $282 billion annually in the U.S. alone (47), leveraging structured, evidence-based PE programs, alongside accessible forms of community PA like cycling, may provide scalable and sustainable opportunities to promote health and resilience in youth during both ordinary and extraordinary times.

The purpose of this study was to expand upon our earlier findings, with focus on understanding the impact of the R4F program in a COVID-19 emergent population. Our previous work demonstrated that the Riding for Focus (R4F) scholastic-based cycling program is associated with improved mental health and well-being in adolescents during the COVID-19 pandemic, providing additional support regarding the importance of cycling as a form of PA (39). Our earlier work showed that participating in the R4F program was associated with enhanced psychosocial well-being and a reduced relative risk of developing psychosocial disorders (12). R4F is a 6–8-week curriculum-based PE program designed for middle school students, providing exposure to and education about cycling activities. In this study, we were able to examine associations and dose-response relationships between lifestyle behaviors and mental health outcomes in a cohort of students post-COVID-19. We hypothesized that participation in the R4F program would be associated with improvements in adolescent students' mental health and well-being, and that these benefits would be evident when considering both modifiable (e.g., physical activity, sleep, screen time) and nonmodifiable (e.g., gender, race/ethnicity, socioeconomic status) risk factors.

## 2 Methods

### 2.1 Participants and methods

The methods for the current study were largely adapted from our previous research (12). Each school's participation in the R4F program is determined by the individual school. In some schools, every student within a grade level will go through the R4F program. In other schools, the program may be within an elective course. Data on students with physical disabilities and special educational needs is not currently collected, but is an area of future interest. However, many schools do report sourcing adaptive bikes to ensure all students are included, and this is an area of future growth and development of the program. The current study lacked a comparison/control group, like our previous study, and instead relied on before and after program participation measurements (12). The program was part of standard educational practice at the school, and parents and legal guardians were sent home letters outlining school participation in the program and surveys as part of the program. Parents or legal guardians who did not wish for their child to participate in the survey signed a form and returned it to the teacher (passive consent). In addition, each student had the option to opt-out of the survey, even if a parent or legal guardian did not opt them out. At the beginning of the surveys, students were invited to only continue if they would like to participate in the surveys, with no impact to their grades. Data were collected via a voluntary anonymous survey that was provided in English as well as Spanish from participants in the Outride R4F scholastic middle school cycling physical education program, further ensuring participant anonymity and minimizing risk. The study was approved by an Institutional Review Board (Advarra), with secondary analysis approved by Loma Linda University IRB. Participants completed online surveys through a Qualtrics platform on classroom or at-home electronic devices before and after the R4F program, which may introduce variability in the survey environment. We do not have information on how much additional instruction was provided to students at each different school or by their parents in the home environment.

**Figure 1:**
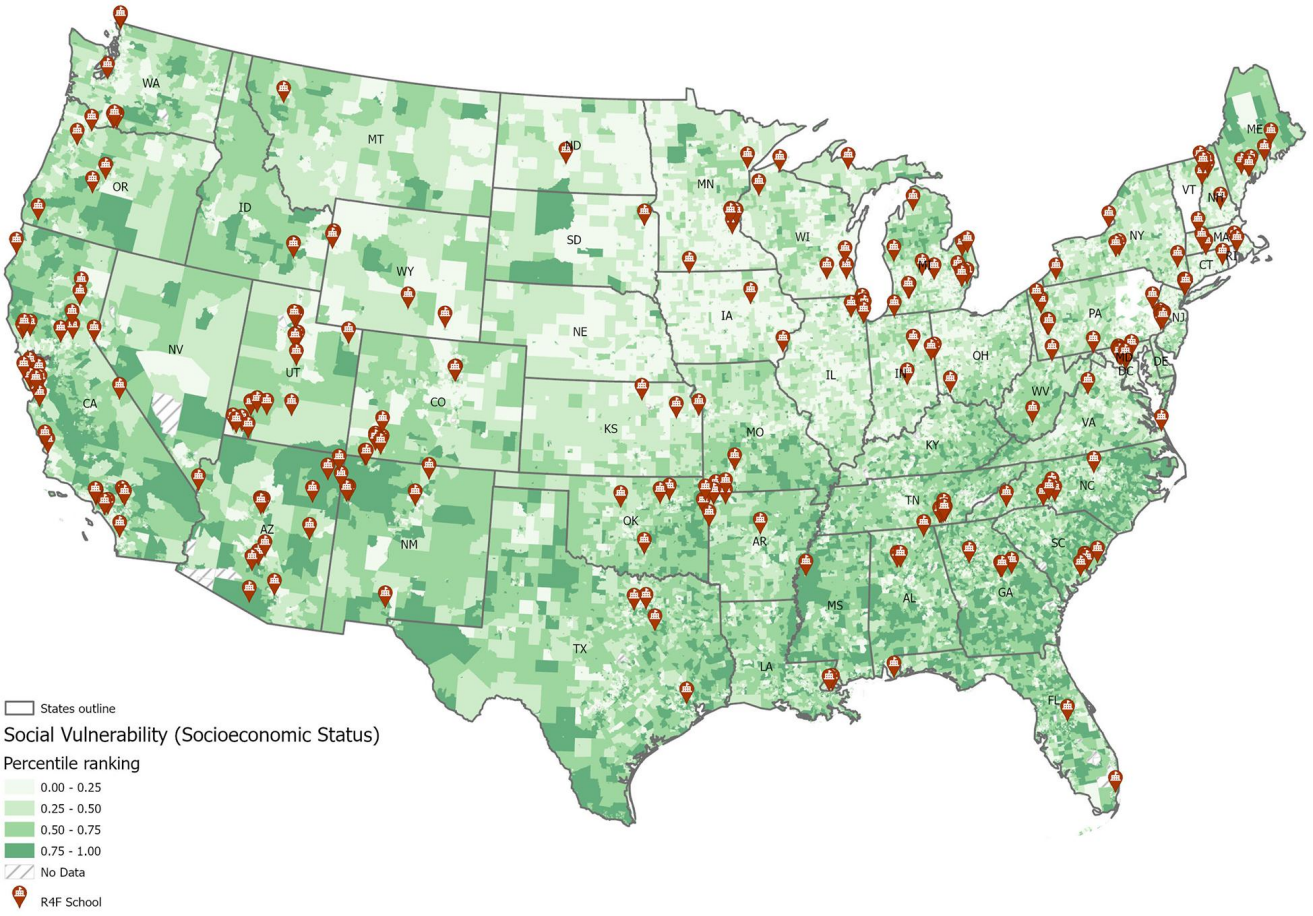
Schools with riding for focus programs are distributed in multiple states and in locations with varied median household incomes.

A total of 8,639 student responses (4,820 PRE surveys and 3,819 POST surveys) from 44 schools across the United States who participated in the R4F program during the 2021–2022 school year participated in the surveys, during a period of easing COVID-19 lockdown restrictions (48). As the surveys were conducted anonymously as part of a program evaluation and surveillance process, we were unable to verify that every student completed both a pre- and post-program survey, with analysis being performed using unpaired analysis techniques. To achieve the most balanced and unbiased set of PRE and POST responses across schools a series of filters were applied to the dataset. In an initial screen, 13 schools that only participated in PRE surveys or POST surveys but not consistently both sets of surveys were removed from analysis, resulting in 8,007 remaining responses (92.7%) from 31 participating schools. To ensure only students who actively completed the survey during class time were included, we also filtered survey responses based on response time. The mean time for survey completion was 10.56 min, with a standard deviation (SD) of 10.27 min. Surveys completed in fewer than 3 min or not completed within 31.1 min (2 SD longer than the mean completion time) were removed. This method was used to provide a more unbiased approach to eliminate data where we suspect that the participant did not perform the survey with intent, including answering the questions too rapidly, without properly reading the question or answer set or that the individual allowed the survey to sit open for many minutes without being attentive to the survey. The length of the survey for the current study is shorter than other routine surveys or assessments taken during the school day, many of which far exceed 10–15 min. For example, the full Youth Risk Behavioral Survey takes about 35 min for students to complete (49), while the California School Climate, Health, and Learning core module takes middle school students an average of 17 min (50). In addition, we applied a response quality filtration, including only surveys with complete responses to all sub-items of the youth self-report version of the Pediatric Symptom Checklist (PSC-17-Y) (51) and WHO-5 well-being index (52, 53). This step prevented skewed scores due to non-responses to individual items and resulted in the removal of an additional 794 survey responses. After applying these filters, the final dataset consisted of 3,924 pre-program survey responses and 3,289 post-program survey responses across 31 schools. Unlike our previous study (12), most schools were not subject to COVID-19 related lockdowns during this period (54, 55). The included 31 schools for this study represent 14% of the total number of schools that have been awarded the R4F program since its inception. Students were between the ages of 11 and 14, corresponding to grade levels 6 through 8, and are considered adolescents as defined by WHO and UN criteria (11) and by modern viewpoints regarding the transition to adulthood (56). The schools included in this study were from multiple regions throughout the United States, as depicted in Figure 1. The demographics of self-reported participant gender, race and ethnicity along with lifestyle and other factors are provided in Table 1.

**Table 1:** Demographic information.

Category	Pre-program survey (N)	Pre %	Post-program survey (N)	Post %
Total Respondents (overall)	3,924	100	3,289	100
Gender				
Female	1,638	42%	1,343	41%
Male	1,853	47%	1,555	47%
A Gender Not Listed	121	3%	121	4%
Prefer Not to Say	312	8%	270	8%
Race and Ethnicity				
American Indian or Alaska Native	77	2.0%	70	2.1%
Asian	353	9.0%	248	7.5%
Black	345	8.8%	301	9.2%
Hispanic	808	20.6%	673	20.5%
Native Hawaiian/Pacific Islander	54	1.4%	31	0.9%
More than One Race	387	9.9%	398	12.1%
White	1,588	40.5%	1,312	39.9%
*Underrepresented Groups (Aggregate)*	*2,024*	*59* *.* *5%*	*1,721*	*60* *.* *1%*
Other Demographic & Lifestyle Factors				
Receive Free/Reduced Lunch—Yes	963	25%	1,127	34%
Receive Free/Reduced Lunch—No	1,066	27%	867	26%
Receive Free/Reduced Lunch—I don't know	1,895	48%	1,295	39%
School Club Engagement—Yes	2,581	66%	2,231	68%
School Club Engagement—No	1,343	34%	1,058	32%
Meets Breakfast Recommendation (7 days/week)	1,560	40%	1,242	38%
Meets Physical Activity Goal (≥4 days/week)	1,006/3,907	26%	911/3,275	28%
Electronic Device Use ≤2 h/day	1,444/3,903	37%	1,111/3,276	34%
Adequate Sleep (≥8.5 h/night)	2,254/3,902	58%	1,826/3,267	56%

Students self-reported their gender, race, and ethnicity. For gender, students self-selected between Male, Female, A gender not listed, and Prefer not to say (Table 1). Due to the small sample sizes of participants not selecting Female or Male, only students who selected Female and Male were included in the comparative analyses. For race and ethnicity, students self-reported as American Indian or Alaska Native, Asian, Black, Hispanic or Latino, Native Hawaiian or Pacific Islander, More than One Race, White, or they did not specify (Table 1). As the data are analyzed using many sub-categories (i.e., days per week physically active), the small sample sizes in many of the reported racial and ethnic minority groups become challenging to interpret. As a result, throughout this manuscript, students from racial and ethnic minority groups (American Indian or Alaska Native, Asian, Black, Hispanic or Latino, Native Hawaiian or Pacific Islander, or students who selected More than One Race) will be aggregated for analysis and referred to as underrepresented groups (UG). While these data were aggregated for statistical power, this grouping may mask distinct experiences across racial and ethnic subgroups.

Students also answered questions about sleep duration, screen time, PA levels, and involvement in clubs and sports teams. They also self-reported their previous participation in a R4F program, and whether they received free or reduced-cost school lunches, which served as a proxy for socioeconomic status (SES) (12, 57). To further understand the SES of the study population, we evaluated the social vulnerability index of each school site, which was 0.41 ± 0.23 (mean ± SD) with a range of 0.10 to 0.92 on a 0–1 scale (58). The schools were in the following SVI ranges, including 8 low SVI, 12 moderate, 2 high, and 4 very high.

To measure psychosocial well-being, students completed assessments that were chosen because they are adolescent centered and designed for a middle school level. Questions were chosen from pre-existing validated instruments for this age group and address both positive and negative psychosocial functioning. Many questions were adopted from CDC's Middle School Youth Risk Behavioral Survey (59). Among the number of metrics available including the Strengths and Difficulties Questionnaire (SDQ) (60), KIDSCREEN (61), the WHO-5 Well-Being Index (52, 53) and the youth self-report version of the PSC-17-Y (51, 62, 63), we chose the WHO-5 and PSC-17-Y metrics based on their psychometric strengths, their brevity, and ease of delivery in school settings. Although these are both strong metrics, it is worth noting concerns regarding the psychometric properties of the WHO-5 and PSC-17-Y measures used in this age group. While they are both established instruments, their validity and sensitivity in younger adolescents (11–14 years) may be limited, as this age range presents distinct developmental characteristics that can influence both self-reflection and reporting accuracy (64, 65). Reliance on self-report alone may also overlook important behavioral or contextual factors; complementing these data with proxy-reports from parents or teachers, as supported by instruments such as the SDQ or KIDSCREEN, could provide a more balanced perspective in future work.

The WHO-5 is a 5-item, self-report questionnaire evaluating positive well-being and depression, validated for use with adolescent respondents ages 11 and older with higher scores indicating greater well-being (66, 67). The PSC-17-Y complements the WHO-5, helping to identify and assess emotional and behavioral problems, with lower scores indicating fewer psychosocial challenges (63, 68). The PSC-17-Y is not meant as a diagnostic tool, rather it is meant to help understand what emotional and behavioral challenges a child may be facing. It covers a range of emotional and behavioral problems and assesses psychosocial functioning, including sub-scores reflecting behavioral conduct and disruptive behavior (externalization), anxiety and depressive symptoms (internalization), and attention difficulties (attention). As both the WHO-5 and PSC-17-Y scores may fluctuate slightly over time, it is important to consider what is considered a meaningful change, as opposed to a statistically significant change. Here, we report WHO-5 scores out of 100, with changes of 10% or more reflecting a meaningful change (69). Each of the 5 items was scored 0 (“At no time”) to 5 (“All of the time”), yielding a raw total of 0–25, which was converted to a percentage score by multiplying by 4. For the PSC each of the17 items were scored 0 (“Never”), 1 (“Sometimes”), or 2 (“Often”) for a total score from 0 to 34. While a difference of 6 or greater for the total score, or differences of 2 or more on the subscales are considered a reliable change for the longer 35-item version (51, 63), reliable change benchmarks have not been published for the short-form version. After completing the R4F program, students took the same survey with additional open and closed questions about the program. The survey was designed to be completed in 10–15 min.

### 2.2 Cycling program curriculum

The Riding for Focus (R4F) program curriculum used in the current study was identical to that employed in our previous research (12). Designed to be integrated into the school day, R4F sessions are run by the physical education teachers at the school and typically take place during students' physical education class time. The program is structured to span 6 to 8 weeks, comprising 12 recommended sessions. Throughout these sessions, students acquire various cycling skills while aiming to achieve at least 20 min of moderate-to-vigorous PA per session. The skills are designed to prepare the participants to be safe while riding on the road. This includes how to start and stop the bike, how to balance and control the bike, along with shifting gears, how to scan and avoid obstacles, and how to conduct themselves as a vehicle on the roadway. Activities are conducted on diverse terrains surrounding the school campus. The R4F program is designed to provide 1 h of PA each day, adhering to the Public Health Strategies (PHS) and WHO guidelines for PA (27, 28). The program also and aligns with the standards set by SHAPE America (70), ensuring a comprehensive and well-rounded approach to physical education and cycling instruction.

### 2.3 Statistical methods

Data analysis procedures and graph production largely followed the methods outlined in our previous study (12). We used R (71) to create data frames and filter data, streamlining the workflow for the current dataset. GraphPad Prism 10 (La Jolla, CA) was employed for graphing and statistical analyses. Prior to analysis, all datasets were tested for normality, with none showing normal distribution. For comparisons between two groups, we used the Mann–Whitney *U*-test to identify potential differences in well-being metrics. For analyses involving more than two groups, we performed a Kruskal–Wallis ANOVA with Dunn's multiple comparisons test. Graphs present violin plots showing median, 25th and 75th quartiles, along with minimum and maximum data ranges. Statistical significance was set at *P* < 0.05, with further distinctions made for *P* < 0.01, *P* < 0.001, and *P* < 0.0001 where appropriate. We calculated Cohen's d test statistic based on Cohen's recommendations (72, 73) to estimate effect size in pre- and post-program comparisons. According to widely accepted benchmarks, a Cohen's d value of 0.2 reflects a small effect size, 0.5 indicates a medium effect, and 0.8 or above signifies a large effect. These thresholds are valuable because they help interpret the real-world significance of observed changes, emphasizing not only whether a difference is statistically significant but also whether it is likely to be meaningful in practice. Consequently, Cohen's d provides additional insight into the practical magnitude of changes in adolescent mental health and well-being scores beyond what traditional *p*-values alone can offer. To examine the relative clinical risk of developing psychosocial disorders, we used a Fisher Exact Test in combination with Koopman asymptotic scores (74) for PSC-17-Y and WHO-5 metrics, based on established cutoff scores of ≥15 and ≤ 50, respectively (63, 67). Decreases in the PSC-17-Y scores signify reduced anxiety, depression, attention, and behavioral challenges, while increases in the WHO-5 score are reflective of better mental health and well-being. Both of these instruments are used as screening tools, and individuals meeting the established cutoff scores are referred to for professional evaluation and potential treatment for mental health disorders.

We evaluated the impact of various risk factors on WHO-5 and PSC-17-Y scores using specific cutoffs for various risk factors based on current research and recommendations as outlined in our previous work (12). The physical activity cutoff was set at 4 days a week of 60 min of PA, as adolescents who do not meet this threshold have an increased risk of developing psychosocial disorders compared to those who participate in PA more (75). For sleep, we used a cutoff of 8 h each weeknight, considering that adolescents who sleep less than the recommended amount (9 h for 6–12 year olds, 8 h for 13–18 year olds) on school nights are at greater risk of psychosocial disorders (76). Screen time was limited to 2 h a day, in line with the American Academy of Pediatrics' 2016 policy statement on adolescent media use (77). Regarding breakfast consumption, we set the cutoff at 7 days a week, as regular breakfast consumption is associated with maintaining a healthy body weight, greater life satisfaction, and better academic performance (78, 79). For breakfast consumption we only analyzed whether something was eaten, without considering the content or caloric amount of the meal.

We conducted linear regression analyses to examine relationships between psychosocial wellness scores (WHO-5 and PSC-17-Y) and various self-reported factors, including hours of sleep, screen time, the number of days of breakfast consumption, and the number of days of PA that met PHS guidelines. An *F*-test was used to determine if the slope of each association was significantly different from zero (80). Additionally, we compared the slopes of the pre- and post-program regression analyses using an *F*-test to assess whether the two datasets shared a common slope or had independent slopes (80). For all slope analyses, a *P*-value of *P* < 0.05 was considered statistically significant.

## 3 Results

**Figure 2:**
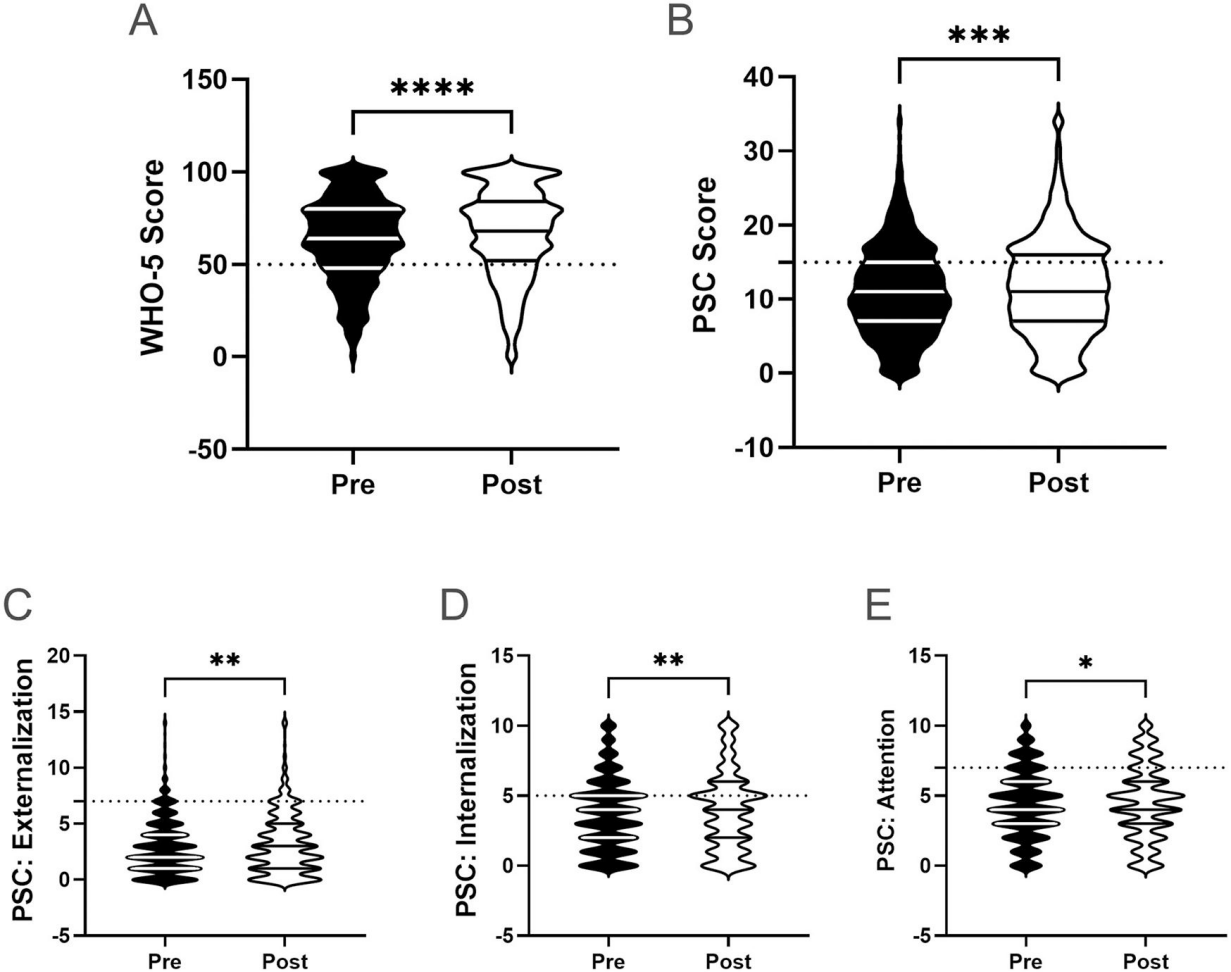
General effectiveness of the R4F program on psychosocial wellness. **(A)** Distribution of WHO-5 well-being scores in a violin plot. Dashed horizontal line shows the critical risk cutoff of 50 points with lower scores indicating lower well-being. **(B)** Distribution of PSC-17-Y composite well-being scores with a critical risk cutoff of 15 points. **(C–E)** PSC-17-Y sub scores of externalization, internalization, or attention on violin plots with critical risk cutoffs of 7 points, 5 points, and 7 points, respectively. Higher scores on the PSC and sub scores indicate lower well-being. Within each violin plot the median and 25% and 75% quartiles are shown. Asterisks denote significant difference between groups based on a Mann–Whitney *U*-test (* < 0.05, ** < 0.01, *** < 0.001, **** < 0.0001). *N* = 3,924 for pre-program, *N* = 3,289 for post-program.

Based on our previous report examining the efficacy of the Riding for Focus (R4F) program on adolescent health and well-being during COVID-19, we aimed to assess whether this effect would be replicated in a cohort of students not subjected to the same restrictions and stresses as our initial study group (3, 12, 16, 19, 20, 54, 55, 81). To evaluate the potential association between the R4F program and mental health and well-being among the student population, we analyzed WHO-5 and PSC-17-Y scores before and after the R4F program. A summary of findings, including statistical test outcomes, can be reviewed in Tables 2, 3. Consistent with our previous findings (12), students' WHO-5 increased by 5% following the R4F program (M_PRE_ = 63.3, M_POST_ = 66.3; Figure 2A). However, we also observed a 5% increase in PSC-17-Y composite scores (M_PRE_ = 11.1, M_POST_ = 11.7) departing from previous findings. Each of the PSC-17-Y sub scores also demonstrated small but significant increases after participation in the R4F program (Table 2): externalization increased by 7% (M_PRE_ = 3.0, M_POST_ = 3.2; Figure 2C), internalization by 5% (M_PRE_ = 3.7, M_POST_ = 3.9; Figure 2D), and attention by 3% (M_PRE_ = 4.4, M_POST_ = 4.5; Figure 2E).

**Table 2:** Results of multivariate analysis.

Category	Pre		Post		Z Statistic	*P*-Value	Sig.	Cohen's *d*	Effect Size
	Mean	SD	Mean	SD					
General	3,924		3,289						
WHO-5	63.3	22.6	66.3	24.0		<0.0001	****	0.1287	Small
PSC-17-Y General	11.1	6.1	11.7	6.6		0.0005	***	0.0944	Small
PSC-17-Y Extern.	3.0	2.5	3.2	2.7		0.0021	**	0.0769	Small
PSC-17-Y Intern.	3.7	2.6	3.9	2.7		0.0048	**	0.0755	Small
PSC-17-Y Attention	4.4	2.4	4.5	2.6		0.0418	*	0.0399	Small
Gender: Male	1,853		1,555						
WHO-5	69.5	20.4	72.6	22.0	5.164	<0.0001	****	0.1461	Small
PSC-17-Y General	10.1	5.9	10.4	6.4	1.542	0.7381	n.s.	0.0487	Small
PSC-17-Y Extern.	3.0	2.5	3.1	2.6	0.590	>0.9999	n.s.	0.0392	Small
PSC-17-Y Intern.	3.1	2.4	3.2	2.6	2.044	0.2459	n.s.	0.0400	Small
PSC-17-Y Attention	4.0	2.4	4.1	2.5	0.6689	>0.9999	n.s.	0.0408	Small
Gender: Female	1,638		1,343						
WHO-5	59.9	22.3	62.6	23.1	3.331	0.0052	**	0.1189	Small
PSC-17-Y General	11.2	5.9	11.9	6.3	3.165	0.0093	**	0.1147	Small
PSC-17-Y Extern.	2.6	2.3	3.0	2.5	3.362	0.0046	**	0.1665	Small
PSC-17-Y Intern.	4.1	2.5	4.3	2.6	1.696	0.5391	n.s.	0.0784	Small
PSC-17-Y Attention	4.4	2.4	4.6	2.5	2.229	0.1549	n.s.	0.0816	Small
Race: White	1,588		1,312						
WHO-5	64.1	22.3	66.0	23.7	2.669	0.0454	*	0.0813	Small
PSC-17-Y General	10.9	6.1	11.5	6.6	2.731	0.0379	*	0.1027	Small
PSC-17-Y Extern.	2.7	2.4	2.9	2.6	1.829	0.4044	n.s.	0.0886	Small
PSC-17-Y Intern.	3.7	2.6	4.0	2.8	2.595	0.0567	n.s.	0.1044	Small
PSC-17-Y Attention	4.4	2.5	4.6	2.6	1.666	0.5744	n.s.	0.0583	Small
Race: UG	2,024		1,721						
WHO-5	63.3	22.5	66.7	24.2	5.341	<0.0001	****	0.147	Small
PSC-17-Y General	11.2	6.1	11.8	6.7	2.196	0.1685	n.s.	0.0842	Small
PSC-17-Y Extern.	3.2	2.6	3.4	2.8	2.041	0.2475	n.s.	0.0856	Small
PSC-17-Y Intern.	3.7	2.5	3.9	2.7	1.648	0.596	n.s.	0.0979	Small
PSC-17-Y Attention	4.3	2.5	4.5	2.6	1.526	0.7620	n.s.	0.0612	Small
SES: Low	963		1,127						
WHO-5	64.4	23.4	67.6	24.4	3.539	0.0024	**	0.1339	Small
PSC-17-Y General	11.3	6.2	11.8	6.8	1.507	0.7915	n.s.	0.0768	Small
PSC-17-Y Extern.	3.1	2.6	3.3	2.7	1.729	0.5030	n.s.	0.0755	Small
PSC-17-Y Intern.	3.8	2.6	3.9	2.8	0.9007	>0.9999	n.s.	0.0370	Small
PSC-17-Y Attention	4.5	2.5	4.6	2.6	1.006	>0.9999	n.s.	0.0392	Small
SES: High	1,066		867						
WHO-5	64.5	22.2	66.6	23.5	2.486	0.0775	n.s.	0.0919	Small
PSC-17-Y General	10.7	6.1	11.4	6.7	2.016	0.2630	n.s.	0.1093	Small
PSC-17-Y Extern.	2.9	2.6	3.1	2.8	1.647	0.5974	n.s.	0.0740	Small
PSC-17-Y Intern.	3.5	2.5	3.9	2.7	2.786	0.0320	*	0.1537	Small
PSC-17-Y Attention	4.2	2.5	4.3	2.6	0.7049	>0.9999	n.s.	0.0392	Small
Breakfast: met	1,560		1,242						
WHO-5	69.5	21.0	73.2	21.2	4.689	<0.0001	****	0.1754	Small
PSC-17-Y General	9.4	5.9	9.8	6.2	2.059	0.2371	n.s.	0.0661	Small
PSC-17-Y Extern.	2.5	2.3	2.7	2.5	2.519	0.0707	n.s.	0.0833	Small
PSC-17-Y Intern.	3.1	2.4	3.2	2.5	0.839	>0.9999	n.s.	0.0408	Small
PSC-17-Y Attention	3.8	2.4	3.9	2.5	1.191	>0.9999	n.s.	0.0408	Small
Breakfast: not met	2,357		2,039						
WHO-5	59.2	22.7	62.0	24.6	4.772	<0.0001	****	0.1183	Small
PSC-17-Y General	12.2	6.0	12.8	6.6	2.329	0.1191	n.s.	0.0951	Small
PSC-17-Y Extern.	3.3	2.5	3.5	2.8	1.608	0.6472	n.s.	0.0754	Small
PSC-17-Y Intern.	4.1	2.6	4.4	2.8	2.532	0.0680	n.s.	0.1110	Small
PSC-17-Y Attention	4.8	2.4	4.9	2.6	1.231	>0.9999	n.s.	0.0400	Small
Sleep: met	2,254		1,826						
WHO-5	68.8	20.6	72.5	21.1	5.529	<0.0001	****	0.1774	Small
PSC-17-Y General	9.7	5.7	10.0	6.1	1.786	0.4447	n.s.	0.0508	Small
PSC-17-Y Extern.	2.6	2.3	2.8	2.5	1.870	0.3685	n.s.	0.0833	Small
PSC-17-Y Intern.	3.2	2.4	3.3	2.5	1.223	>0.9999	n.s.	0.0408	Small
PSC-17-Y Attention	3.9	2.3	3.9	2.5	0.748	>0.9999	n.s.	0.0000	Small
Sleep: not met	1,648		1,441						
WHO-5	55.7	23.0	58.3	25.2	3.879	0.0006	***	0.1078	Small
PSC-17-Y General	13.0	6.2	13.7	6.7	2.580	0.0593	n.s.	0.1084	Small
PSC-17-Y Extern.	3.5	2.7	3.8	2.9	1.976	0.2886	n.s.	0.1071	Small
PSC-17-Y Intern.	4.5	2.7	4.7	2.8	2.418	0.0937	n.s.	0.0727	Small
PSC-17-Y Attention	5.0	2.4	5.2	2.6	1.762	0.4682	n.s.	0.0799	Small
Physical Activity: met	1,006		911						
WHO-5	71.4	21.9	73.8	23.7	5.017	0.0257	*	0.1052	Small
PSC-17-Y General	10.2	6.4	10.5	7.0	2.823	>0.9999	n.s.	0.0447	Small
PSC-17-Y Extern.	2.9	2.7	3.0	2.8	2.447	>0.9999	n.s.	0.0364	Small
PSC-17-Y Intern.	3.2	2.5	3.3	2.8	2.372	>0.9999	n.s.	0.0377	Small
PSC-17-Y Attention	4.1	2.5	4.1	2.7	1.557	>0.9999	n.s.	0.0000	Small
Physical Activity: not met	2,901		2,364						
WHO-5	60.5	22.1	63.4	23.6	3.083	<0.0001	****	0.1268	Small
PSC-17-Y General	11.4	6.0	12.1	6.5	2.447	0.0010	***	0.1119	Small
PSC-17-Y Extern.	3.0	2.4	3.3	2.6	2.200	0.0060	**	0.1199	Small
PSC-17-Y Intern.	3.9	2.6	4.2	2.7	1.958	0.0093	**	0.1132	Small
PSC-17-Y Attention	4.5	2.4	4.7	2.5	1.703	0.0843	n.s.	0.0816	Small
Screentime: met	1,444		1,111						
WHO-5	69.4	21.3	71.7	22.6	3.095	0.0118	*	0.1047	Small
PSC-17-Y General	9.5	5.8	10.1	6.5	2.356	0.1109	n.s.	0.0974	Small
PSC-17-Y Extern.	2.5	2.3	2.8	2.5	2.076	0.2273	n.s.	0.1249	Small
PSC-17-Y Intern.	3.2	2.4	3.4	2.6	1.417	0.9395	n.s.	0.0799	Small
PSC-17-Y Attention	3.8	2.4	3.9	2.6	1.780	0.4502	n.s.	0.0400	Small
Screentime: not met	2,459		2,165						
WHO-5	59.9	22.5	63.5	24.3	6.229	<0.0001	****	0.1537	Small
PSC-17-Y General	12.0	6.1	12.5	6.6	1.857	0.3799	n.s.	0.0787	Small
PSC-17-Y Extern.	3.2	2.5	3.4	2.7	1.757	0.4731	n.s.	0.0769	Small
PSC-17-Y Intern.	4.1	2.6	4.2	2.7	1.956	0.3025	n.s.	0.0377	Small
PSC-17-Y Attention	4.7	2.4	4.8	2.6	0.514	>0.9999	n.s.	0.0400	Small
Extracurricular Participation: Yes	2,581		2,231						
WHO-5	65.6	22.1	68.0	23.4	4.100	0.0002	***	0.1055	Small
PSC-17-Y General	10.8	6.1	11.6	6.6	4.121	0.0002	***	0.1259	Small
PSC-17-Y Extern.	2.9	2.5	3.2	2.7	3.664	0.0015	**	0.1153	Small
PSC-17-Y Intern.	3.6	2.5	3.9	2.7	3.356	0.0048	**	0.1153	Small
PSC-17-Y Attention	4.3	2.5	4.5	2.6	2.661	0.0468	*	0.0784	Small
Extracurricular Participation: No	1,343		1,058						
WHO-5	58.8	23.0	62.8	25.0	4.900	<0.0001	****	0.1665	Small
PSC-17-Y General	11.6	6.1	11.7	6.7	0.279	>0.9999	n.s.	0.0156	Small
PSC-17-Y Extern.	3.1	2.5	3.2	2.6	0.203	>0.9999	n.s.	0.0392	Small
PSC-17-Y Intern.	4.0	2.7	4.0	2.8	0.289	>0.9999	n.s.	0.0000	Small
PSC-17-Y Attention	4.5	2.4	4.5	2.6	0.183	>0.9999	n.s.	0.0000	Small

Asterisks found in the “Sig”. column denote significance difference between pre and post for the aggregate data (general) based on a Mann–Whitney *U-*test (* < 0.05, ** < 0.01, *** < 0.001, **** < 0.0001). For all subsequent groups, a Kruskal–Wallis One-way ANOVA with a Dunn's multiple comparison test based on ranks (* < 0.05, ** < 0.01, *** < 0.001, **** < 0.0001) was used. SES, socioeconomic status. Metrics include the World Health Organization-5 (WHO-5), the pediatric symptom checklist (PSC-17-Y), and its subsequent subscores of externalization (PSC-17-Y Extern.), internalization (PSC-17-Y Intern.) and attention (PSC-17-Y Attention).

**Table 3:** Pairwise comparisons within risk factors across pre both and post surveys.

Measure	Survey Type	Level 1	Level 2	Z	Adjusted *p*-value
WHO-5	Pre	Female	Male	12.42	<0.0001
WHO-5	Post	Female	Male	12.78	<0.0001
PSC-17-Y	Pre	Female	Male	5.35	<0.0001
PSC-17-Y	Post	Female	Male	6.57	<0.0001
WHO-5	Pre	UG Students	White Students	1.89	0.35, *n.s.*
WHO-5	Post	UG Students	White Students	.75	>0.999*, n.s.*
PSC-17-Y	Pre	UG Students	White Students	2.62	0.053, *n.s.*
PSC-17-Y	Post	UG Students	White Students	1.43	0.92, *n.s.*
WHO-5	Pre	Low SES	High SES	.08	>0.999, *n.s.*
WHO-5	Post	Low SES	High SES	1.00	>0.999, *n.s.*
PSC-17-Y	Pre	Low SES	High SES	2.37	0.11, *n.s.*
PSC-17-Y	Post	Low SES	High SES	1.76	0.47, *n.s.*
WHO-5	Pre	Sleep: Met	Sleep: Not Met	17.4	<0.0001
WHO-5	Post	Sleep: Met	Sleep: Not Met	16.97	<0.0001
PSC-17-Y	Pre	Sleep: Met	Sleep: Not Met	16.51	<0.0001
PSC-17-Y	Post	Sleep: Met	Sleep: Not Met	16.23	<0.0001
WHO-5	Pre	Physical Activity: Met	Physical Activity: Not Met	13.63	<0.0001
WHO-5	Post	Physical Activity: Met	Physical Activity: Not Met	12.59	<0.0001
PSC-17-Y	Pre	Physical Activity: Met	Physical Activity: Not Met	8.98	<0.0001
PSC-17-Y	Post	Physical Activity: Met	Physical Activity: Not Met	8.62	<0.0001
WHO-5	Pre	Screentime: Met	Screentime: Not Met	12.52	<0.0001
WHO-5	Post	Screentime: Met	Screentime: Not Met	9.62	<0.0001
PSC-17-Y	Pre	Screentime: Met	Screentime: Not Met	12.72	<0.0001
PSC-17-Y	Post	Screentime: Met	Screentime: Not Met	10.36	<0.0001
WHO-5	Pre	Breakfast: Met	Breakfast: Not Met	13.61	<0.0001
WHO-5	Post	Breakfast: Met	Breakfast: Not Met	13.29	<0.0001
PSC-17-Y	Pre	Breakfast: Met	Breakfast: Not Met	14.05	<0.0001
PSC-17-Y	Post	Breakfast: Met	Breakfast: Not Met	12.53	<0.0001
WHO-5	Pre	Extracurricular Participation	No Extracurricular Participation	8.88	<0.0001
WHO-5	Post	Extracurricular Participation	No Extracurricular Participation	5.79	<0.0001
PSC-17-Y	Pre	Extracurricular Participation	No Extracurricular Participation	3.62	0.0018
PSC-17-Y	Post	Extracurricular Participation	No Extracurricular Participation	.37	>0.999, *n.s.*

UG Students, students from underrepresented groups; SES, socioeconomic status; Met, met recommendation; Not Met, did not meet recommendation. *P*-Values have been adjusted for multiple comparisons. Means and S.D. for each group are reported in Table 2.

The relative clinical risk for the development of mental health disorders was assessed by examining the number of respondents who either met or did not meet critical clinical cutoff scores for both the WHO-5 (score ≤ 50) and the PSC-17-Y (score ≥ 15) (12, 63, 67). Participation in the R4F program was associated with a reduced relative risk (.91, 95% CI [0.84, 0.99), as measured by the WHO-5 in all study participants. In comparison, the relative risk as determined by the PSC-17-Y was higher post-program (1.17, 95% CI [1.1, 1.26).

### 3.1 Analysis of specific risk factors and their interplay with the R4F program and daily recommendations

#### 3.1.1 Gender

 (12) and others have demonstrated that both psychosocial well-being and responses to PA programs often differ across gender (82–86). In line with this work, we examined whether there were gender differences in mental well-being, as well as whether any potential differences changed after participation in the R4F program. We also assessed whether there were differences in the relative risk of poor mental health across genders.

**Figure 3:**
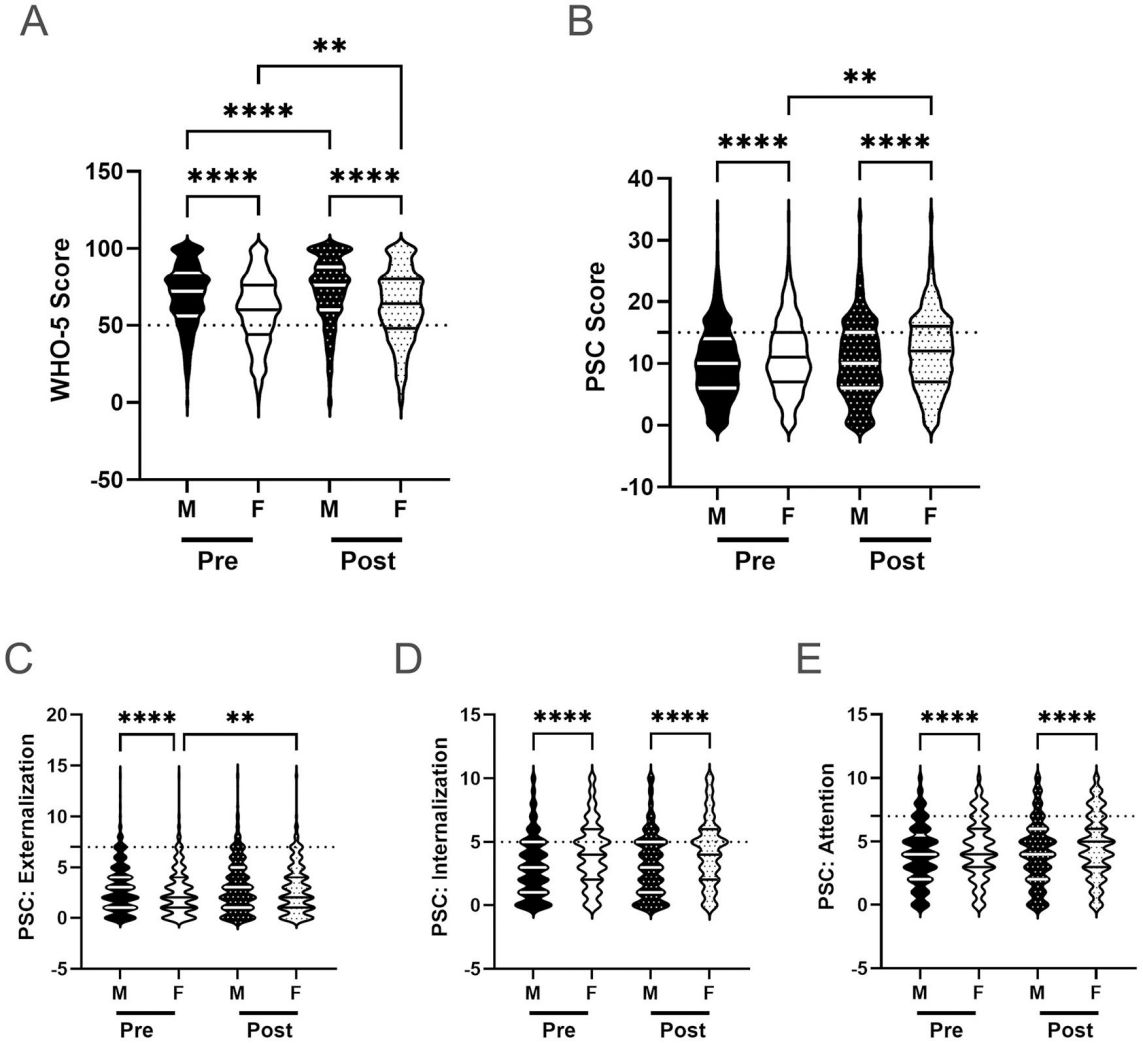
Gender and the effectiveness of the R4F program on psychosocial wellness. **(A)** Distribution of WHO-5 well-being scores in a violin plot. Dashed horizontal line shows the critical risk cutoff of 50 points with lower scores indicating lower well-being. **(B)** Distribution of PSC-17-Y composite well-being scores with a critical risk cutoff of 15 points. **(C–E)** PSC-17-Y sub scores of externalization, internalization, or attention on violin plots with critical risk cutoffs of 7 points, 5 points, and 7 points, respectively. Higher scores indicate lower well-being. Within each violin plot the median and 25% and 75% quartiles are shown. Asterisks denote significant difference between groups based on a Kruskal–Wallis One-way ANOVA with a Dunn's multiple comparison test based on ranks (** < 0.01, **** < 0.0001). Pre-program, *N* = 1,853 for male, *N* = 1,638 for female. Post-program, *N* = 1,555 for male, *N* = 1,343 for female.

Prior to the implementation of the R4F program, we observed differences in psychosocial well-being scores across gender. Males demonstrated higher WHO-5 scores (M = 69.5) compared to females (M = 59.9), indicating better overall well-being (Figure 3A). Females also showed higher PSC-17-Y composite scores (M = 11.2) than males (M = 10.1), reporting more psychosocial challenges (Figure 3B). When examining the PSC-17-Y sub scores, males exhibited higher externalization scores (M_Male_ = 3.0, M_Female_ = 2.6; Figure 3C), while females scored higher in both internalization (M_Male_ = 3.1, M_Female_ = 4.1) and attention (M_Male_ = 4.0, M_Female_ = 4.4) domains (Figures 3D,E).

After completing the R4F program, both male and female students showed improvements in their WHO-5 scores, with males experiencing a 5% increase (M_PRE_ = 69.5, M_POST_ = 72.6) and females a 5% increase (M_PRE_ = 59.9, M_POST_ = 62.6) compared to before the program (Figure 3A). The gap between males and females persisted post-program. Regarding the PSC-17-Y, male students' scores did not change after completing the program, while female students' scores increased by 6% (M_PRE_ = 11.2, M_POST_ = 11.9; Figure 3B).

Examining the PSC-17-Y sub scores, males showed no changes in any of the three domains following the program (Figures 3C–E). In contrast, females experienced a 15% increase in their externalization scores (M_PRE_ = 2.6, M_POST_ = 3.0; Figure 3C), resulting in no significant difference between males and females in this domain post-program. Females' internalization and attention sub scores remained unchanged after the R4F program (Figures 3D,E). Notably, females maintained significantly higher internalization and attention sub scores compared to males, regardless of program participation.

**Figure 4:**
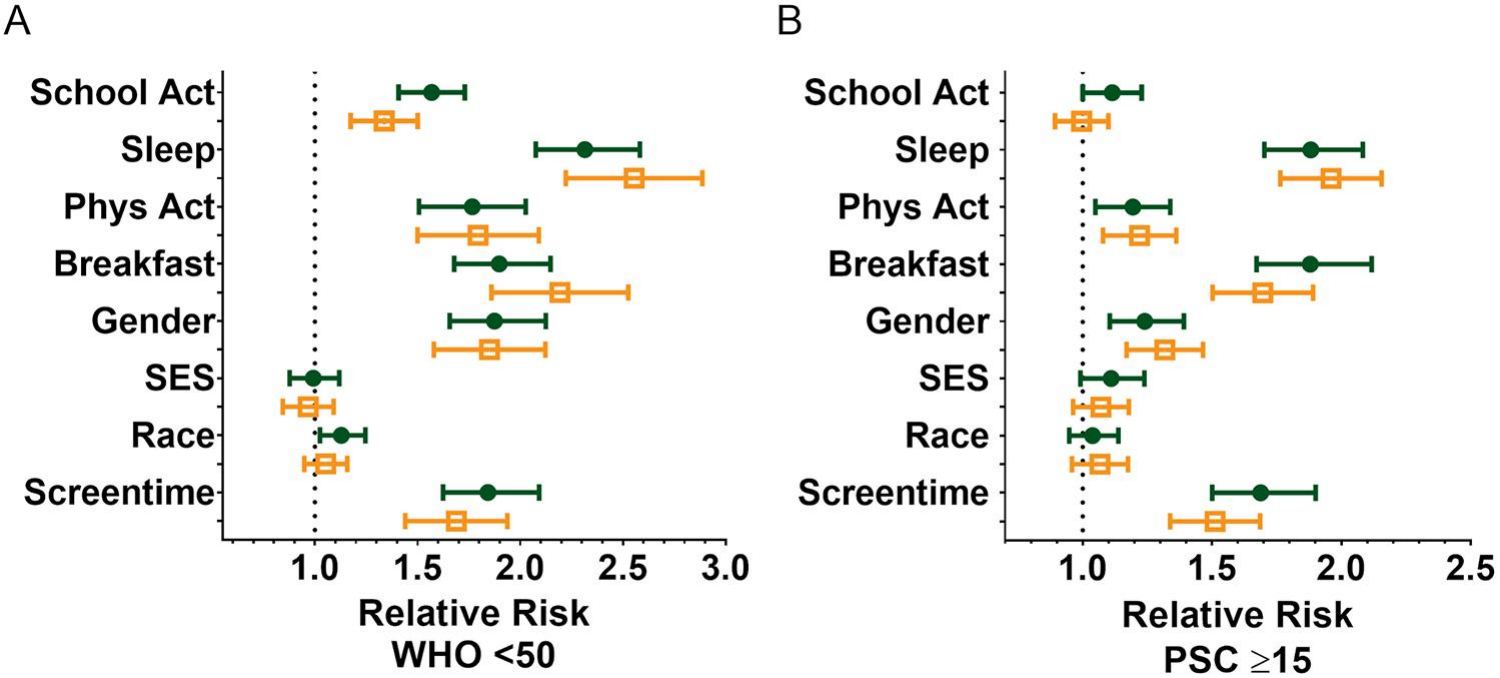
Impact of the R4F program on the relative risk of developing psychosocial disorders. **(A)** Relative risk based on WHO-5 scores below the critical cutoff of 50. **(B)** Relative risk based on PSC-17-Y scores 15 and above. Shown are the mean +/− 95CI before (green circles) and after (orange open squares) the program. As indicated on the figure, relative risk was performed for the influence of being involved in school activity **(Y)** vs. uninvolved in school activity **(N)** (School Activity), ≥8 h of sleep/night vs. <8 h of sleep/night (Sleep), ≥4 days/week of PA vs. <4 days/week of PA (Physical Activity), eating breakfast 7 days/week <7 days/week (Breakfast), being male vs. female (Gender), low-income vs. medium/high-income (SES), White identifying **(W)** vs. Underrepresented Groups (UG), ≤2 h/day screen time vs. >2 h/day of screen time (Screen time), and based on performing a Koopman asymptotic score (74).

We also assessed the relative risk of male and female students developing psychosocial illnesses using both the WHO-5 (Figure 4A) and the PSC-17-Y (Figure 4B). Female students were found to be at a higher relative risk for developing mental health disorders than male students across both metrics before (WHO-5: 1.9, 95% CI [1.7,2.1]; PSC: 1.2, 95% CI [1.1, 1.4) and after (WHO-5: 1.8, 95% CI [1.6,2.1]; PSC: 1.3, 95% CI [1.2, 1.5) the R4F program.

#### 3.1.2 Race and ethnicity

Previous research has indicated that students from underrepresented groups typically report lower scores of mental well-being, compared to their White peers (12, 87, 88). Here, we assessed whether there are differences in self-reported psychosocial well-being across students from racial and ethnic minority backgrounds and White students. We also examined whether any potential differences were affected by participation in the R4F program.

**Figure 5:**
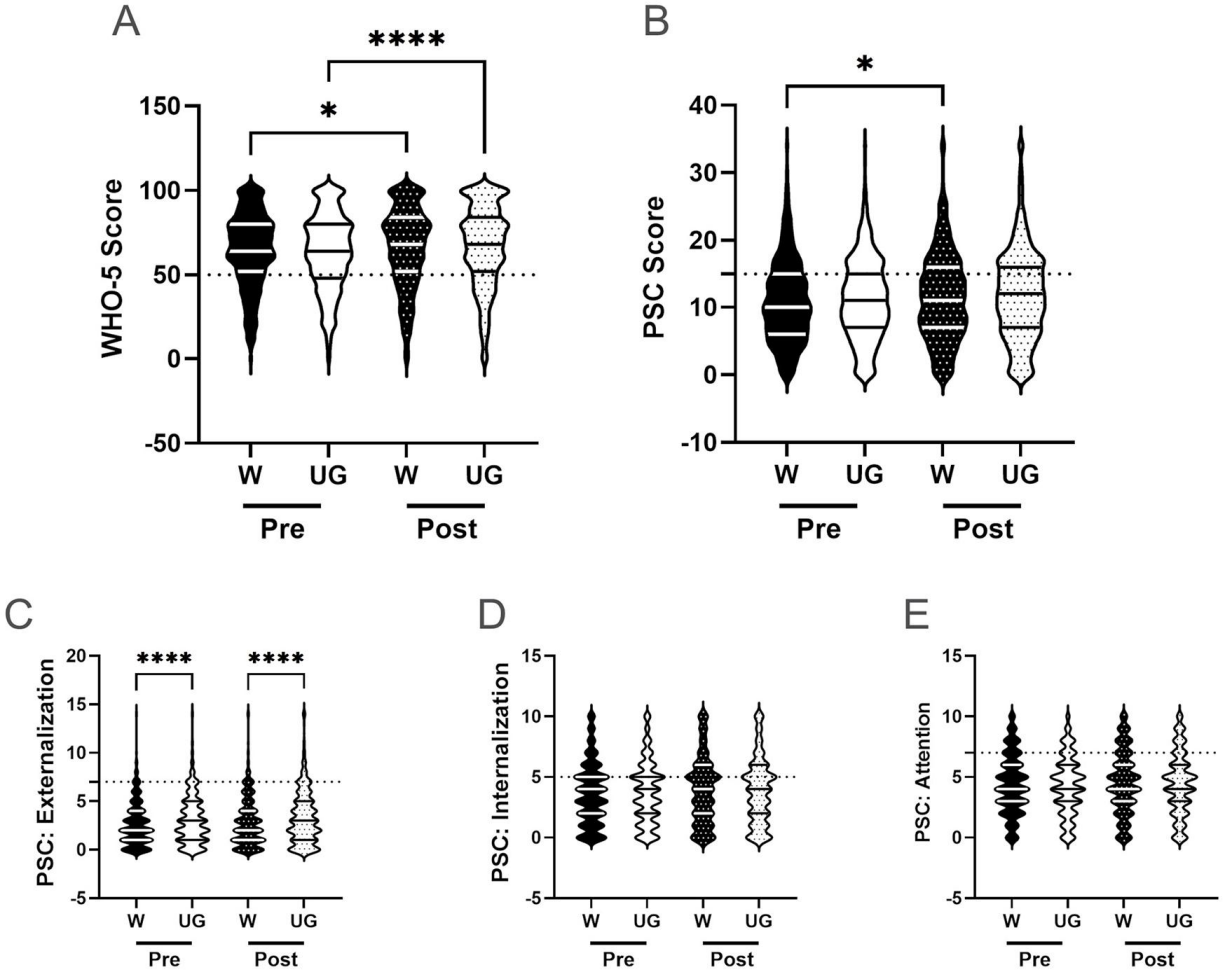
Racial identity and the effectiveness of the R4F program on psychosocial wellness. **(A)** Distribution of WHO-5 well-being scores in a violin plot. Dashed horizontal line shows the critical risk cutoff of 50 points with lower scores indicating lower well-being. **(B)** Distribution of PSC-17-Y composite well-being scores with a critical risk cutoff of 15 points. **(C–E)** PSC-17-Y sub scores of externalization, internalization, or attention on violin plots with critical risk cutoffs of 7 points, 5 points, and 7 points, respectively. Higher scores indicate lower well-being. Within each violin plot the median and 25% and 75% quartiles are shown. Asterisks denote significant difference between groups based on a Kruskal–Wallis One-way ANOVA with a Dunn's multiple comparison test based on ranks (* < 0.05, **** < 0.0001). W = White students, UG = Underrepresented Groups. Pre-program, *N* = 1,588 for white students, *N* = 2,024 for Underrepresented Groups. Post-program, *N* = 1,312 for white students, *N* = 1,721 for Underrepresented Groups.

Before participating in the R4F program, both students from underrepresented groups and White students showed similar scores on most measures of psychosocial well-being, including WHO-5 composite scores (M_UG_ = 63.3, M_W_ = 64.1; Figure 5A), PSC-17-Y composite scores (M_UG_ = 11.2, M_W_ = 10.9; Figure 5B), and PSC-17-Y internalization (M_UG_ = 3.7, M_W_ = 3.7; Figure 5D) and attention sub scores (M_UG_ = 4.3, M_W_ = 4.4; Figure 5E). However, students from underrepresented groups had significantly higher externalization sub scores (M = 3.2) compared to White students (M = 2.7) prior to the program (Figure 5C).

Upon completion of the R4F program, both groups showed improvements in WHO-5 composite scores, with White students increasing by 3% (M_PRE_ = 64.1, M_POST_ = 66.0) and students from underrepresented groups by 5% (M_PRE_ = 63.3, M_POST_ = 66.7; Figure 5A). There were no significant differences between the two groups either before or after the program. White students' PSC-17-Y composite scores increased following the program by 6% (M_PRE_ = 10.9, M_POST_ = 11.5) while the scores of students from underrepresented groups remained unchanged (Figure 5B). No significant differences were observed between the groups after program participation.

Examining the PSC-17-Y sub scores post-program, both students from underrepresented groups and White students' sub scores showed no significant changes following the program (externalization, internalization, and attention; Figures 5C–E). The externalization sub scores of students from underrepresented groups remained elevated relative to White students after the program (M_UG_ = 3.4, M_W_ = 2.9; Figure 5C).

The relative risk of developing a psychosocial illness was assessed using critical clinical cutoff scores for both the WHO-5 (Figure 4A) and the PSC-17-Y (Figure 4B). Students from underrepresented groups had similar risks of poor mental well-being compared to White students before the R4F program (WHO-5: 1.1, 95% CI [1.0,1.2]; PSC: 1.0, 95% CI [0.95, 1.1) as well as following the program as delineated by the WHO-5 metric (1.0, 95% CI [0.95, 1.2) and PSC-17-Y metric (1.1, 95% CI [0.96, 1.2).

#### 3.1.3 Socioeconomic status

We next examined the role of family socioeconomic status (SES) on adolescents' psychosocial well-being, as numerous studies have reported associations between these factors (84, 89–91). Consistent with our previous methodology, we used students' self-reported qualification for free or reduced lunch as a proxy measure for SES (12). This approach allowed us to investigate potential differences in psychosocial outcomes based on socioeconomic background within the context of the R4F program.

**Figure 6:**
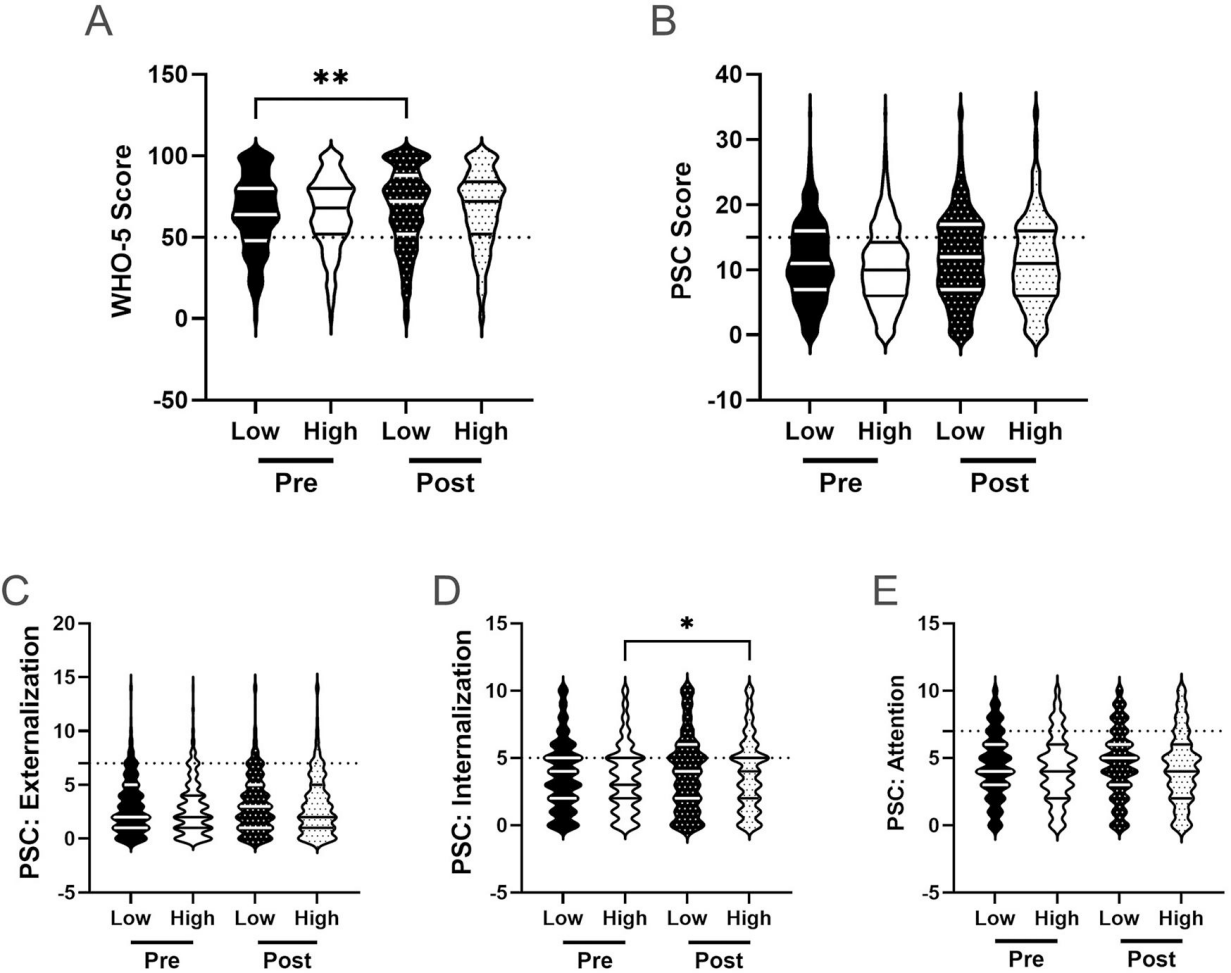
Socioeconomic status and the effectiveness of the R4F program on psychosocial wellness. **(A)** Distribution of WHO-5 well-being scores in a violin plot. Dashed horizontal line shows the critical risk cutoff of 50 points with lower scores indicating lower well-being. **(B)** Distribution of PSC-17-Y composite well-being scores with a critical risk cutoff of 15 points. **(C–E)** PSC-17-Y sub scores of externalization, internalization, or attention on violin plots with critical risk cutoffs of 7 points, 5 points, and 7 points, respectively. Higher scores indicate lower well-being. Within each violin plot the median and 25% and 75% quartiles are shown. Asterisks denote significant difference between groups based on a Kruskal–Wallis One-way ANOVA with a Dunn's multiple comparison test based on ranks (* < 0.05, ** < 0.01). H = intermediate/high SES, L = low SES. Pre-program *N* = 963 for low SES, *N* = 1,066 for intermediate/high SES. Post-program, *N* = 1,127 for low SES, *N* = 867 for intermediate/high SES.

Prior to the R4F program, there were no significant differences between students of different socioeconomic status (SES) on their WHO-5 composite scores (Figure 6A), PSC-17-Y composite scores (Figure 6B), or any of the PSC-17-Y sub scores (externalization, internalization, and attention; Figures 6C–E). Following the R4F program, students who qualified for free or reduced lunch (low SES) showed a significant 5% increase in WHO-5 scores (M_PRE_ = 64.4, M_POST_ = 67.6), while the difference in high SES students' scores was not significant (M_PRE_ = 64.5, M_POST_ = 66.6; Figure 6A). There were no significant differences in WHO-5 scores between low and high SES groups, regardless of program participation. PSC-17-Y composite scores remained similar between the two SES groups both before and after the R4F program (Figure 6B).

Examining the PSC-17-Y sub scores, participation in the R4F program was not associated with changes in externalization and attention sub scores for either SES group, which remained similar between groups (Figures 6C,E). Internalization sub scores did not differ from pre to post program for low SES students (M_PRE_ = 3.8, M_POST_ = 3.9) but increased by 11% for high SES students (M_PRE_ = 3.5, M_POST_ = 3.9; Figure 6D). Despite this change, internalization sub scores were similar between SES groups both before and after the program.

There were no differences in the relative risk for either the WHO-5 or PSC-17-Y metrics both before (WHO-5: 1.0, 95% CI [.9,1.1]; PSC: 1.1, 95% CI [1.0, 1.2) and after the R4F program (WHO-5: 1.0, 95% CI [.8,1.1]; PSC: 1.1, 95% CI [1.0, 1.2), with low and high SES students having equivalent risk.

#### 3.1.4 Club involvement

Previous research has demonstrated that participation in extracurricular activities can have positive effects on mental health and well-being (12, 92). We therefore investigated how students' involvement in at least one extracurricular activity at school (such as sports or music) may influence student mental health outcomes, and whether that interacts with participation in the R4F program. This allowed us to understand whether the R4F program's impact differed between students who were already engaged in other school activities and those who were not.

**Figure 7:**
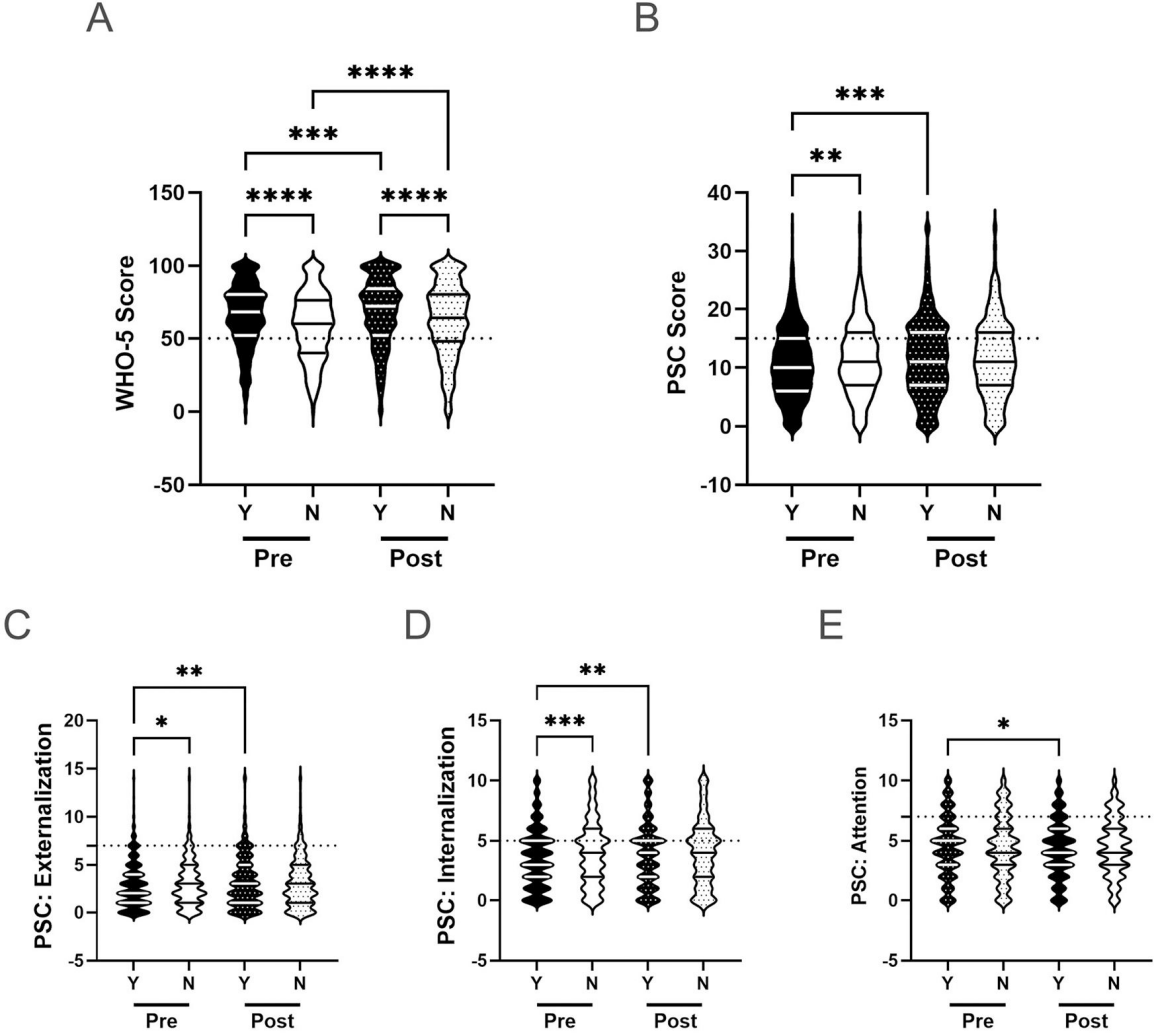
Participation in school activities and the effectiveness of the R4F program on psychosocial wellness. **(A)** Distribution of WHO-5 well-being scores in a violin plot. Dashed horizontal line shows the critical risk cutoff of 50 points with lower scores indicating lower well-being. **(B)** Distribution of PSC-17-Y composite well-being scores with a critical risk cutoff of 15 points. **(C–E)** PSC-17-Y sub scores of externalization, internalization, or attention on violin plots with critical risk cutoffs of 7 points, 5 points, and 7 points, respectively. Higher scores indicate lower well-being. Within each violin plot the median and 25% and 75% quartiles are shown. Asterisks denote significant difference between groups based on a Kruskal–Wallis One-way ANOVA with a Dunn's multiple comparison test based on ranks (* < 0.05, ** < 0.01, *** < 0.001, **** < 0.0001). Y = Involved, N = not involved. Pre-program, *N* = 2,581 for involved, *N* = 1,343 for uninvolved. Post-program, *N* = 2,231 for involved, *N* = 1,058 for uninvolved.

Prior to the R4F program, students involved in clubs demonstrated better psychosocial well-being compared to those without club involvement. Club-involved students had 12% higher WHO-5 scores (M_EC_ = 65.6, M_NoEC_ = 58.8; Figure 7A) and 7% lower PSC-17-Y scores (M_EC_ = 10.8, M_NoEC_ = 11.6; Figure 7B) than students who were not involved in extracurricular clubs. They also had significantly lower externalization and internalization sub scores, by 6% (M_EC_ = 2.9, M_NoEC_ = 3.1) and 10% M_EC_ = 3.6, M_NoEC_ = 4.0) respectively (Figures 7C,D). Attention sub scores were similar between the two groups (Figure 7E).

Following the R4F program, both groups showed improvements in WHO-5 scores, with club-involved students increasing by 4% (M_PRE_ = 65.6, M_POST_ = 68.0) and non-involved students by 7% (M_PRE_ = 58.8, M_POST_ = 62.8; Figure 7A). Club-involved students maintained higher WHO-5 scores both before (12% higher) and after (8% higher) the program than non-involved students. Club participation was associated with a 7% increase in PSC-17-Y scores (M_PRE_ = 10.8, M_POST_ = 11.6), while non-involved students' scores remained unchanged (M_PRE_ = 11.6, M_POST_ = 11.7; Figure 7B). This resulted in similar PSC-17-Y composite scores between the two groups post-program. Examining PSC-17-Y sub scores, club-involved students showed significant increases across all domains: 10% in externalization (M_PRE_ = 2.9, M_POST_ = 3.2; Figure 7C), 8% in internalization (M_PRE_ = 3.6, M_POST_ = 3.9; Figure 7D), and 5% in attention (M_PRE_ = 4.3, M_POST_ = 4.5; Figure 7E). In contrast, non-involved students' sub scores remained unchanged. After the R4F program, there were no significant differences in any sub score measures between the two groups.

With regards to relative risk, before the R4F program, the WHO-5 (1.6, 95% CI [1.4, 1.7), but not PSC-17-Y metric (1.1, 95% CI [1.0, 1.2), indicated that those students not involved in any extracurricular activities were at greater risk of a psychosocial disorder. Following the R4F program, students not involved in an extracurricular activity exhibited reduced relative risk as delineated by the WHO-5 metric (1.3, 95% CI [1.2, 1.5) and were not at-risk as measured by the PSC-17-Y metric (1.0, 95% CI [.9, 1.1).

#### 3.1.5 Breakfast

CDC guidelines recommend that adolescents eat breakfast every day of the week to optimize mental health and well-being (93, 94). To better understand the relationship between breakfast consumption and student mental health, we examined students' self-reported breakfast eating habits before and after program participation. By investigating this factor, we aimed to explore how regular breakfast consumption might interact with the R4F program's effects on students' psychosocial outcomes. In addition, we examined whether there was a dose-response relationship between frequency of eating breakfast and mental well-being.

**Figure 8:**
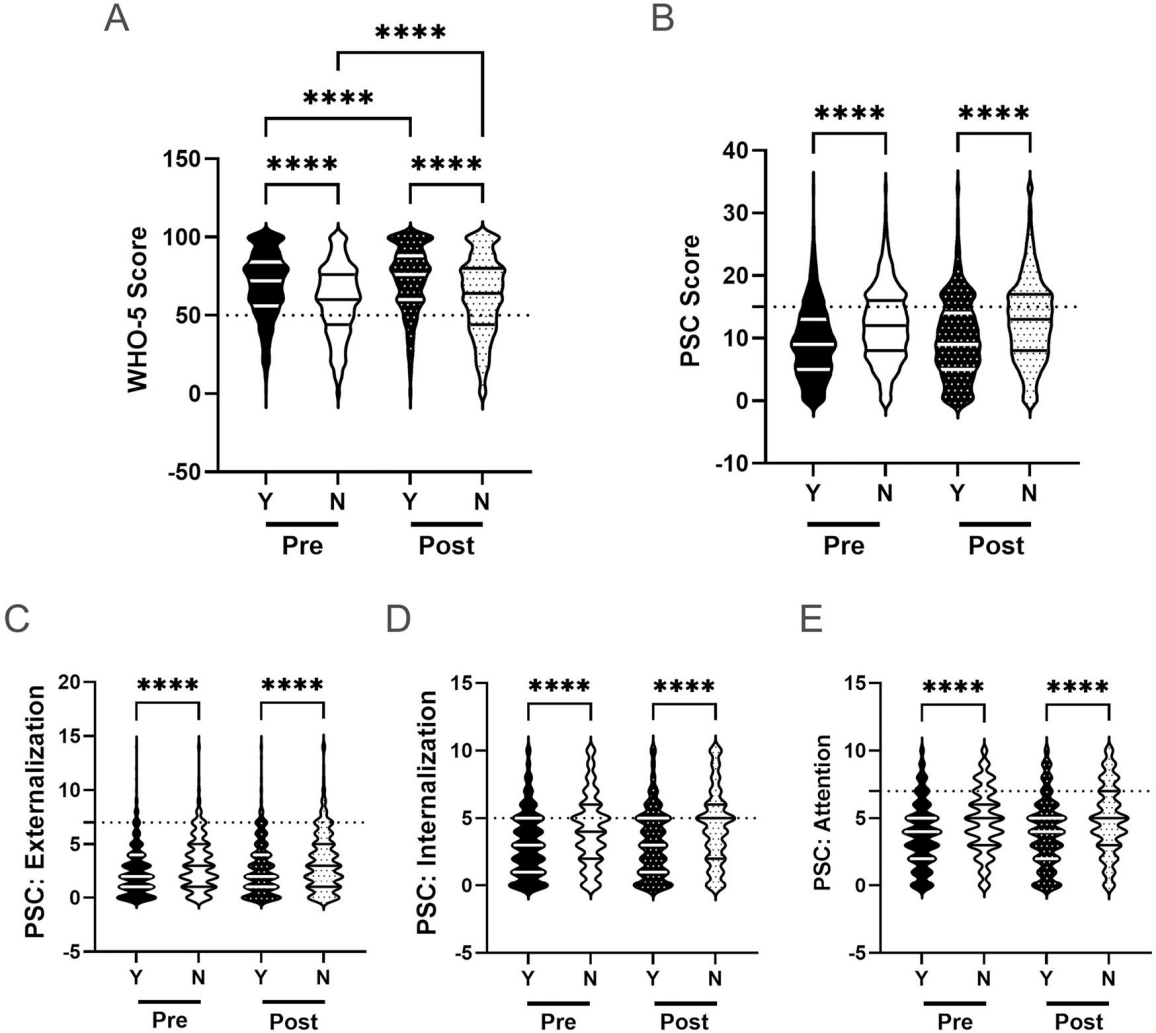
Impact of eating breakfast and the effectiveness of the R4F program on psychosocial wellness. **(A)** Distribution of WHO-5 well-being scores in a violin plot. Dashed horizontal line shows the critical risk cutoff of 50 points with lower scores indicating lower well-being. **(B)** Distribution of PSC-17-Y composite well-being scores with a critical risk cutoff of 15 points. **(C–E)** PSC-17-Y sub scores of externalization, internalization, or attention on violin plots with critical risk cutoffs of 7 points, 5 points, and 7 points, respectively. Within each violin plot the median and 25% and 75% quartiles are shown. Asterisks denote significant difference between groups based on a Kruskal–Wallis One-way ANOVA with a Dunn's multiple comparison test based on ranks (**** < 0.0001). Pre-program, *N* = 1,560 for 7 days/week, *N* = 2,357 for <7 days/week. Post-program, *N* = 1,242 for 7 days/week, *N* = 2,039 for <7 days/week.

Before the R4F program, students who met daily breakfast recommendations demonstrated significantly better psychosocial well-being compared to those who did not, illustrated in the WHO-5 and PSC-17-Y metrics (Table 2). They had 17% higher WHO-5 scores (M_BK_ = 69.5, M_NoBK_ = 59.2; Figure 8A) and 23% lower PSC-17-Y scores (M_BK_ = 9.4, M_NoBK_ = 12.2; Figure 8B). Additionally, their PSC-17-Y sub scores were significantly lower: 24% for externalization (M_BK_ = 2.5, M_NoBK_ = 3.3; Figure 8C), 32% for internalization (M_BK_ = 3.1, M_NoBK_ = 4.1; Figure 8D), and 26% for attention (M_BK_ = 3.8, M_NoBK_ = 4.8; Figures 8E).

After completing the R4F program, both groups showed a 5% increase in WHO-5 scores (Figure 8A), with students meeting breakfast recommendations maintaining an 18% higher score (M = 73.2) than those who did not meet recommendations (M = 62.0). PSC-17-Y composite scores did not change for either group (Figure 8B) with the gap between students who met breakfast recommendations (M = 9.8) and those who did not (M = 12.8) persisting. Externalization, internalization, and attention sub scores also remained unchanged for both groups (Figures 8C–E), with students meeting breakfast recommendations continuing to show significantly lower sub scores than those who did not meet recommendations by 25%, 37%, and 26%, respectively.

Relative risk was also influenced by regular breakfast consumption. Across both metrics, before (WHO-5: 1.9, 95% CI [1.7,2.1]; PSC: 1.9, 95% CI [1.7, 2.1) and after R4F participation (WHO-5: 2.2, 95% CI [1.9,2.5]; PSC: 1.7, 95% CI [1.5, 1.9), students who did not meet breakfast recommendations had higher rates of poor mental health and were at a higher relative risk for developing a psychosocial disorder compared to those who met recommendations (Figures 4A,B).

**Figure 9:**
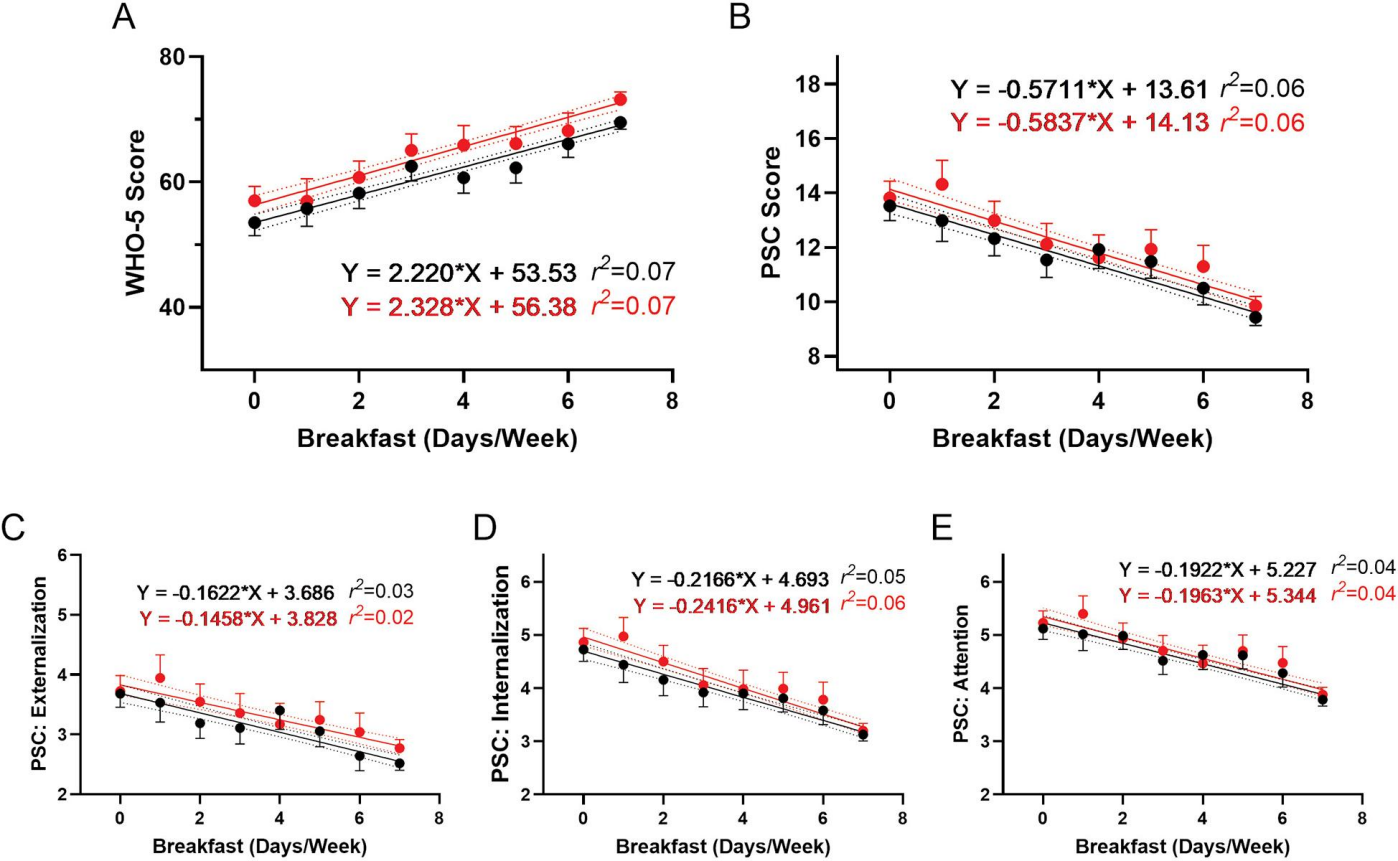
Association of eating breakfast and the effectiveness of the R4F program on psychosocial wellness. **(A)** WHO-5 well-being scores. **(B)** PSC-17-Y composite well-being scores. **(C–E)** PSC-17-Y sub scores of externalization, internalization, or attention. Black dots and lines and for pre-program; Red dots and lines are for post-program. Shown are the mean +/− 95CI, a linear curve fit (solid lines) and 95CI for the linear regression analysis (dotted lines) between the WHO-5 or PSC-17-Y scores and the number of days the participants ate breakfast each week. Also shown are the regression formulae and *r^2^* value for each regression analysis.

To further understand the role of breakfast consumption on psychosocial well-being, we examined the association between the number of days an adolescent ate breakfast and their psychosocial wellness scores. Figure 9A shows the positive association between WHO-5 scores and the frequency of breakfast consumption. Conversely, Figures 9B–E demonstrate negative associations between breakfast frequency and PSC-17-Y composite and sub scores, indicating better well-being with more frequent breakfast consumption. These associations remained consistent following the R4F program, as the slopes of the relationships were not significantly modified by program participation.

#### 3.1.6 Physical activity

The impact of PA on mental health is best established in adult populations, with causality in children and adolescents remaining unresolved (12, 95–97). Towards this end, we examined the role of self-reported students' PA frequency and its effects on psychosocial well-being and whether there was an interrelationship with the R4F program.

**Figure 10:**
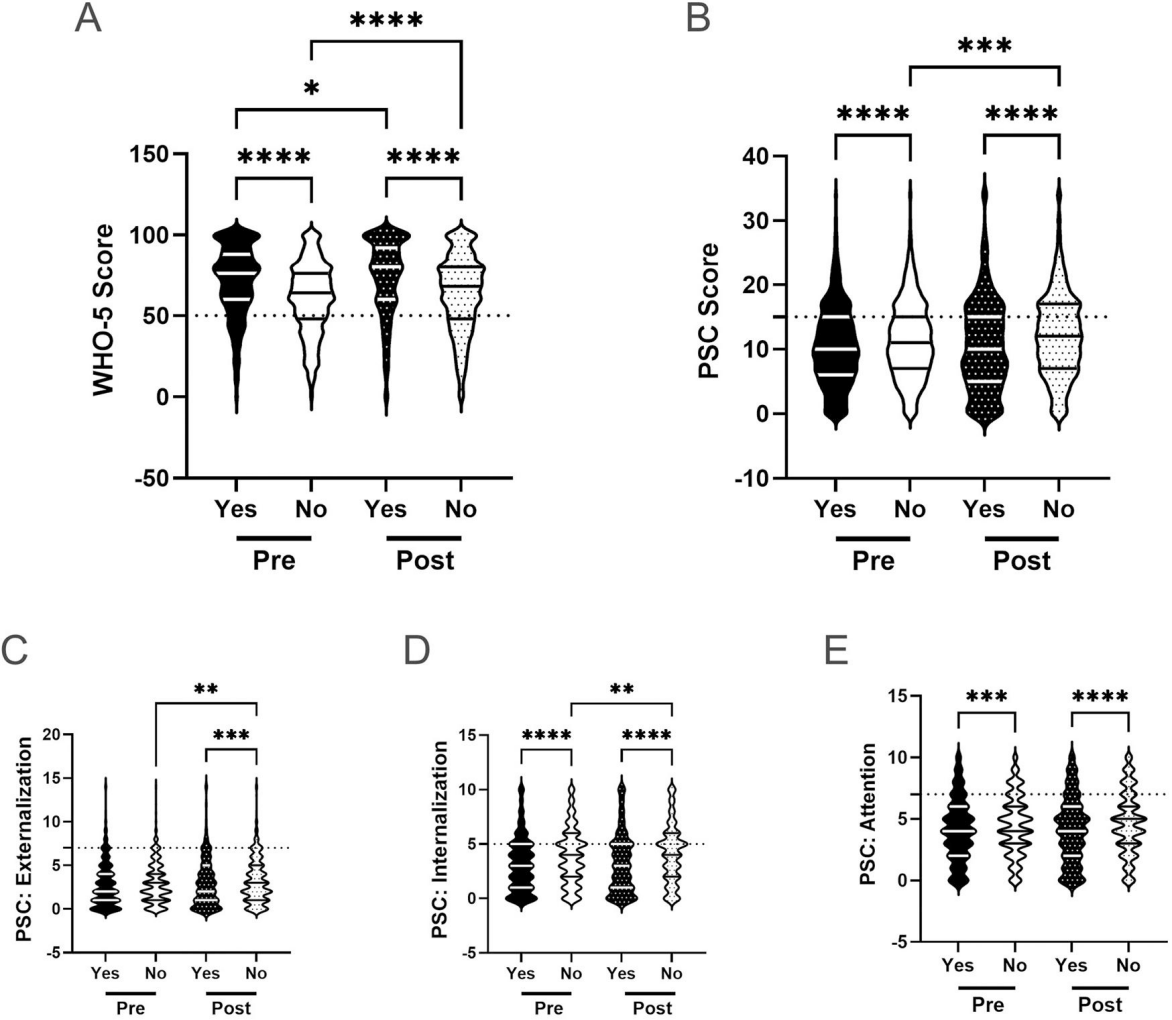
Physical activity and the effectiveness of the R4F program on psychosocial wellness. **(A)** Distribution of WHO-5 well-being scores in a violin plot. Dashed horizontal line shows the critical risk cutoff of 50 points with lower scores indicating lower well-being. **(B)** Distribution of PSC-17-Y composite well-being scores with a critical risk cutoff of 15 points. **(C–E)** PSC-17-Y sub scores of externalization, internalization, or attention on violin plots with critical risk cutoffs of 7 points, 5 points, and 7 points, respectively. Higher scores indicate lower well-being. Within each violin plot the median and 25% and 75% quartiles are shown. Asterisks denote significant difference between groups based on a Kruskal–Wallis One-way ANOVA with a Dunn's multiple comparison test based on ranks (* < 0.05, **** < 0.0001). Pre-program, *N* = 1,006 for ≥4 days/week, *N* = 2,901 for <4 days/week. Post-program, *N* = 911 for ≥4 days/week, *N* = 2,364 for <4 days/week.

Prior to the R4F program, adolescents who participated in PA 4 or more days per week demonstrated 15% higher WHO-5 scores (M = 71.4) compared to those who did not meet this threshold (M = 60.5; Figure 10A). They also showed significantly lower PSC-17-Y composite scores by 12% (M_PA_ = 10.2, M_NoPA_ = 11.4), as well as PSC-17-Y sub scores (externalization, internalization, and attention; Figures 10B–E) by 4% (M_PA_ = 2.9, M_NoPA_ = 3.0), 22% (M_PA_ = 3.2, M_NoPA_ = 3.9), and 9% (M_PA_ = 4.1, M_NoPA_ = 4.5), respectively (Table 2).

Following the R4F program, WHO-5 scores increased for both groups: 3% for students meeting PA recommendations (M_PRE_ = 71.4, M_POST_ = 73.8) and 5% for those who did not (M_PRE_ = 60.5, M_POST_ = 63.4; Figure 10A). Post program, students exercising at least 4 days weekly had 14% higher WHO-5 scores than those who did not. PSC-17-Y composite scores increased by 3% for students who met PA recommendations (M_PRE_ = 10.2, M_POST_ = 10.5) and were increased by 6% for those who did not (M_PRE_ = 11.4, M_POST_ = 12.1; Figure 10B). Post-program, students meeting activity guidelines had 16% lower PSC-17-Y scores than those who did not. PSC-17-Y sub scores showed no differences after the program compared to before the program for both groups, (Figures 10C–E) with students who met PA recommendations continuing to have lower externalization, internalization, and attention sub scores than those who did not meet recommendations by 9%, 26%, and 13%, respectively (Table 2).

The relative risk of developing a psychosocial illness, assessed using critical clinical cutoff scores for both the WHO-5 (Figure 4A) and PSC-17-Y (Figure 4B), indicated that students not meeting PA recommendations consistently were at higher risk for developing psychosocial disorders, both before (WHO-5: 1.8, 95% CI [1.5,2.0]; PSC: 1.2, 95% CI [1.1, 1.3) and after the R4F program (WHO-5: 1.8, 95% CI [1.5,2.1]; PSC: 1.2, 95% CI [1.1, 1.4).

**Figure 11:**
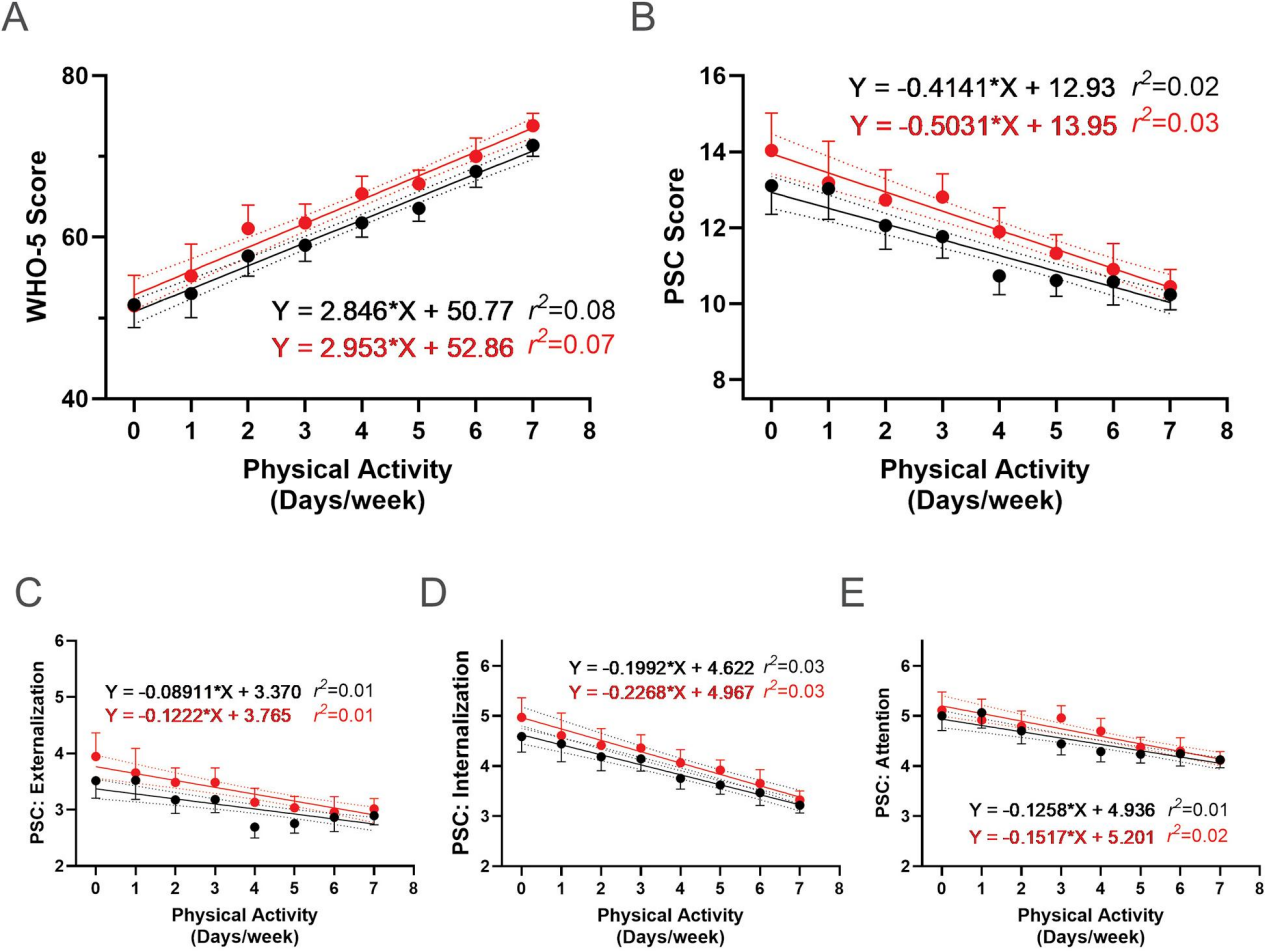
Association between physical activity and the effectiveness of the R4F program on psychosocial wellness. **(A)** WHO-5 well-being scores. **(B)** PSC-17-Y composite well-being scores. **(C–E)** PSC-17-Y sub scores of externalization, internalization, or attention. Black dots and lines and for pre-program; Red dots and lines are for post-program. Shown are the mean +/− 95CI, a linear curve fit (solid lines) and 95CI for the linear regression analysis (dotted lines) between the WHO-5 or PSC-17-Y scores and the number of days participants met physical activity guidelines each week. Also shown are the regression formulae and *r^2^* value for each regression analysis.

Further analysis of the association between PA frequency and psychosocial well-being scores revealed a positive correlation with WHO-5 scores (Figure 11A) and negative correlations with PSC-17-Y composite and sub scores (Figure 11B–E), both before and after the R4F program. These relationships indicate that more frequent PA is associated with better psychosocial well-being outcomes.

#### 3.1.7 Screen time

**Figure 12:**
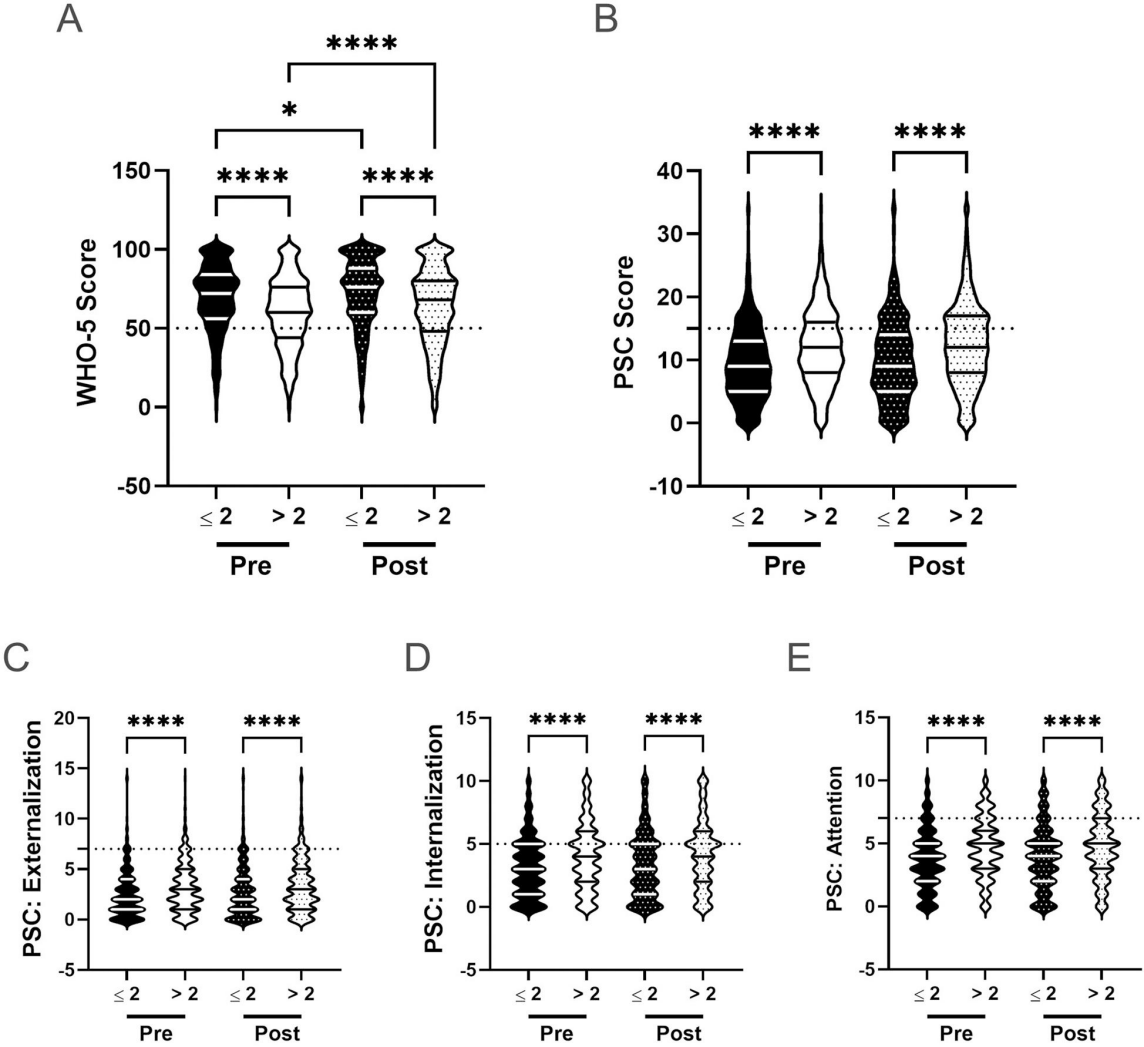
Screen time and the effectiveness of the R4F program on psychosocial wellness. **(A)** Distribution of WHO-5 well-being scores in a violin plot. Dashed horizontal line shows the critical risk cutoff of 50 points with lower scores indicating lower well-being. **(B)** Distribution of PSC-17-Y composite well-being scores with a critical risk cutoff of 15 points. **(C–E)** PSC-17-Y sub scores of externalization, internalization, or attention on violin plots with critical risk cutoffs of 7 points, 5 points, and 7 points, respectively. Higher scores indicate lower well-being. Within each violin plot the median and 25% and 75% quartiles are shown. Asterisks denote significant difference between groups based on a Kruskal–Wallis One-way ANOVA with a Dunn's multiple comparison test based on ranks (* < 0.05, **** < 0.0001). Pre-program, *N* = 1,444 for ≤2 h/day, *N* = 2,459 for >2 h/day. Post-program, *N* = 1,111 for ≤2 h/day, *N* = 2,165 for >2 h/day.

Current literature suggests that spending more than 2 h a day on electronic devices negatively impacts psychosocial well-being (12, 98). We examined the influence of screen time on student well-being, along with whether this changes after participation in the R4F program. Before the R4F program, students who adhered to the 2 h screen time recommendation demonstrated better psychosocial well-being, with significantly higher WHO-5 scores (M_ST_ = 69.4, M_NoST_ = 59.9) and significantly lower PSC-17-Y composite scores (M_ST_ = 9.5, M_NoST_ = 12.0) by 13% and 27%, respectively, compared to those exceeding 2 h a day (Figures 12A,B). Students exceeding the screen time recommendation also had significantly higher baseline externalization, internalization, and attention sub scores by 22% (M_ST_ = 2.5, M_NoST_ = 3.2), 21% (M_ST_ = 3.2, M_NoST_ = 4.1), and 20% (M_ST_ = 3.8, M_NoST_ = 4.7), respectively (Figures 12C–E).

Following the R4F program, both groups showed improvements in WHO-5 scores: a 3% increase for those meeting the screen time recommendation (M_PRE_ = 69.4, M_POST_ = 71.7) and a 6% increase for those exceeding it (M_PRE_ = 59.9, M_POST_ = 63.5; Figure 12A). Students adhering to the 2 h limit maintained 11% higher WHO-5 scores than those who did not meet the recommendation after the program. Both groups showed no change in PSC-17-Y scores, with students who adhered to screen time recommendations maintaining significantly lower PSC-17-Y scores (M_PRE_ = 9.5, M_POST_ = 10.1), by 24%, compared to those that did not (M_PRE_ = 12.0, M_POST_ = 12.5; Figure 12B). There were no significant changes in externalization, internalization, or attention sub scores for either group following the R4F program (Figures 12C–E), with students exceeding screen time recommendations maintaining significantly higher sub scores after the program by 24%, 26%, and 22%, respectively (Table 2).

The relative risk of developing a psychosocial illness, assessed using critical clinical cutoff scores for both the WHO-5 and PSC-17-Y, indicated that students exceeding screen time recommendations were consistently at higher risk for developing psychosocial disorders, both before (WHO-5: 1.8, 95% CI [1.6,2.1]; PSC: 1.7, 95% CI [1.5, 1.9) and after the R4F program (WHO-5: 1.7, 95% CI [1.4,1.9]; PSC: 1.5, 95% CI [1.3, 1.7).

**Figure 13:**
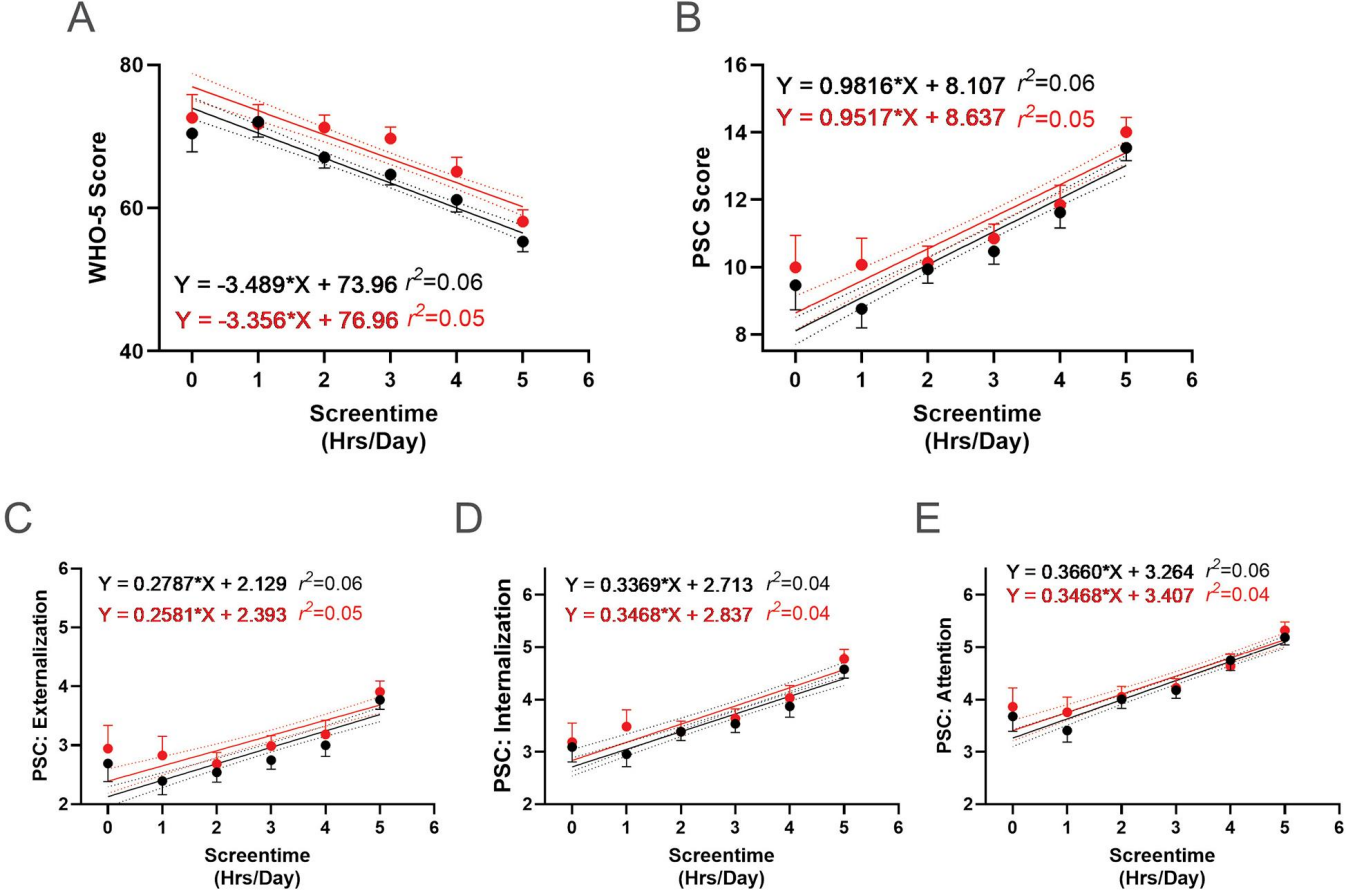
Association between screen time and the effectiveness of the R4F program on psychosocial wellness. **(A)** WHO-5 well-being scores. **(B)** PSC-17-Y composite well-being scores. **(C–E)** PSC-17-Y sub scores of externalization, internalization, or attention. Black dots and lines and for pre-program; Red dots and lines are for post-program. Shown are the mean +/− 95CI, a linear curve fit (solid lines) and 95CI for the linear regression analysis (dotted lines) between the WHO-5 or PSC-17-Y scores and the number of screentime hours participants had each day. Also shown are the regression formulae and *r^2^* value for each regression analysis.

Further analysis revealed a negative association between screen time use and WHO-5 scores, with fewer hours of screen time correlating with higher WHO-5 scores (Figure 13A). Conversely, there was a positive association between screen time use and PSC-17-Y composite and sub scores (Figures 13B–E), with fewer hours correlating with lower scores. These associations remained consistent following the R4F program, as the slopes of the relationships were not significantly altered by program participation.

#### 3.1.8 Sleep

**Figure 14:**
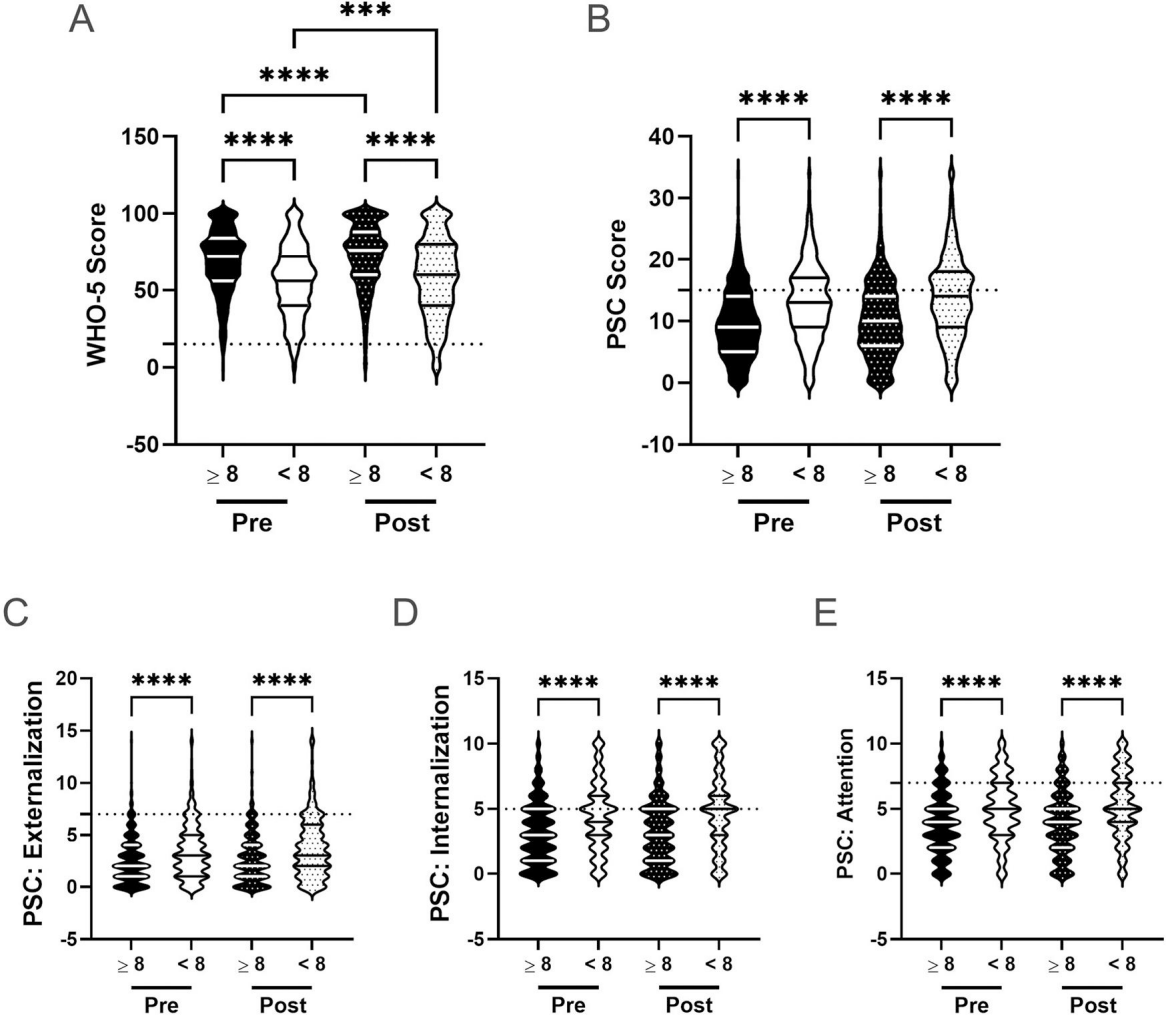
Impact of sleep and the effectiveness of the R4F program on psychosocial wellness. **(A)** Distribution of WHO-5 well-being scores in a violin plot. Dashed horizontal line shows the critical risk cutoff of 50 points with lower scores indicating lower well-being. **(B)** Distribution of PSC-17-Y composite well-being scores with a critical risk cutoff of 15 points. **(C–E)** PSC-17-Y sub scores of externalization, internalization, or attention on violin plots with critical risk cutoffs of 7 points, 5 points, and 7 points, respectively. Higher scores indicate lower well-being. Within each violin plot the median and 25% and 75% quartiles are shown. Asterisks denote significant difference between groups based on a Kruskal–Wallis One-way ANOVA with a Dunn's multiple comparison test based on ranks (*** < 0.001, **** < 0.0001). Pre-program, *N* = 2,254 for sleeping ≥ 8 h/night, *N* = 1,648 for sleeping < 8 h/night. Post-program, *N* = 1,826 for sleeping ≥ 8 h/night, *N* = 1,441 for sleeping < 8 h/night.

Current literature suggests that sleeping 8 or more hours a night positively impacts psychosocial well-being (12, 99, 100). We assessed whether meeting sleep recommendations positively influences students well-being, and whether this is impacted by participating in the R4F program. Before the R4F program, students meeting sleep recommendations demonstrated better psychosocial well-being, with 19% higher WHO-5 scores (M_S_ = 68.8, M_NoS_ = 55.7; Figure 14A) and 34% lower PSC-17-Y scores (M_S_ = 9.7, M_NoS_ = 13.0; Figure 14B) compared to those not meeting recommendations. They also showed significantly lower baseline externalization, internalization, and attention sub scores by 36% (M_S_ = 2.6, M_NoS_ = 3.5), 38% (M_S_ = 3.2, M_NoS_ = 4.5), and 29% (M_S_ = 3.9, M_NoS_ = 5.0), respectively (Figures 14C–E).

Following the R4F program, both groups experienced a 5% increase in WHO-5 scores (Figure 14A), with students meeting sleep recommendations maintaining 20% higher scores (M_PRE_ = 68.8, M_POST_ = 72.5) than those who did not meet recommendations (M_PRE_ = 55.7, M_POST_ = 58.3). PSC-17-Y composite scores remained unchanged for both groups (Figure 14B), with students not meeting sleep recommendations (M_PRE_ = 13.0, M_POST_ = 13.7) consistently showing significantly lower scores post program than those who met sleep recommendations (M_PRE_ = 9.7, M_POST_ = 10.0). There were no significant changes in PSC-17-Y sub scores for either group (Figures 14C–E), but these sub scores remained lower in students meeting sleep recommendations, being 37% for externalization, 42% for internalization, and 33% for attention (Table 2).

The relative risk of developing a psychosocial illness, assessed using critical clinical cutoff scores for both the WHO-5 (Figure 4A) and PSC-17-Y (Figure 4B), indicated that students not meeting sleep recommendations were consistently at higher risk for developing psychosocial disorders, both before (WHO-5: 2.3, 95% CI [2.1,2.6]; PSC: 1.9, 95% CI [1.7, 2.1) and after the R4F program (WHO-5: 2.5, 95% CI [2.2,2.9]; PSC: 2.0, 95% CI [1.8, 2.1).

**Figure 15:**
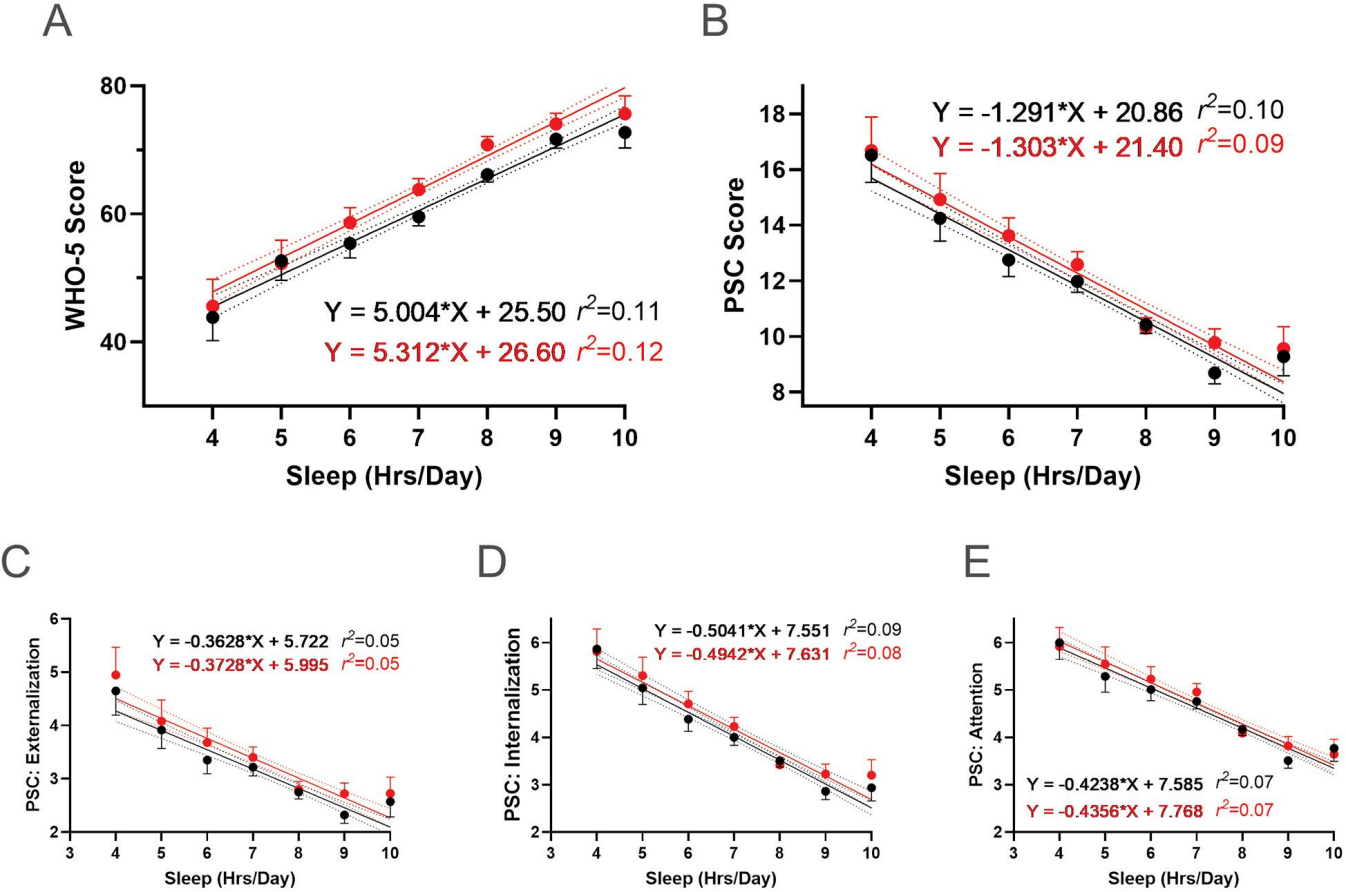
Association between sleep and the effectiveness of the R4F program on psychosocial wellness. **(A)** WHO-5 well-being scores. **(B)** PSC-17-Y composite well-being scores. **(C–E)** PSC-17-Y sub scores of externalization, internalization, or attention. Black dots and lines are for pre-program; Red dots and lines are for post-program. Shown are the mean +/− 95CI, a linear curve fit (solid lines) and 95CI for the linear regression analysis (dotted lines) between the WHO-5 or PSC-17-Y scores and the number of hours of sleep participants had each night. Also shown are the regression formulae and *r^2^* value for each regression analysis.

We also examined whether there were dose-response relationships between sleep and student well-being. There was a positive association between sleep duration and WHO-5 scores, with more hours of sleep a night correlating with higher WHO-5 scores (Figure 15A). Conversely, there was a negative association between sleep duration and PSC-17-Y composite and sub scores (Figures 15B–E), with more hours of sleep correlating with lower scores, indicating better well-being. These associations remained consistent following the R4F program, as the slopes of the relationships were not significantly altered by program participation.

## 4 Discussion

The results of our study on the Riding for Focus (R4F) program in a post-COVID cohort of adolescents reveal a complex picture of its impact on mental health and well-being. Our findings both align with and diverge from our previous research, highlighting the multifaceted nature of adolescent mental health interventions. This topic holds great significance as we have seen poor mental health symptoms become more evident post-COVID-19 pandemic (2–5).

The study revealed persistent gender disparities in adolescent mental health outcomes, both before and after the Riding for Focus (R4F) program, highlighting the complex interplay of these factors (12). Additionally, mental health disparities were observed in students who did not meet recommendations for breakfast consumption, PA, sleep duration, and screen time. Further, these modifiable factors had a dose-dependent impact on student mental well-being, with incremental improvements in both WHO-5 and PSC-17-Y scores for each additional day or hour closer to the recommendation. These findings underscore the need for tailored interventions to address diverse student populations effectively, considering multiple lifestyle factors that impact adolescent well-being.

Similar to our earlier study, we observed a modest increase in WHO-5 composite scores following program participation, which is associated with an improvement in overall well-being. However, unlike our previous findings (12), we also observed small increases in PSC-17-Y scores, suggesting that participants may exhibit a potential decline in some aspects of psychosocial functioning. These contrasting results warrant careful interpretation (63, 68). Importantly, although the changes in both the WHO-5 and PSC-17-Y scores from pre- to post-program are statistically significant, their biological or clinical significance remains uncertain due to the modest magnitude of these changes. In comparison to the current studies, we previously observed much larger, dose-dependent improvements when participants met various lifestyle recommendations. This suggests that the modest shifts seen in the current analysis may not be directly attributable to participation in the program. Instead, they could reflect natural variation over time or other unrelated factors. To clarify the true impact of program participation, further research is needed. These future studies should include appropriate comparison groups to help distinguish between changes that occur naturally and those that result specifically from involvement in the program.

There were also differences between the study populations in the current study and our previous work that may have influenced our findings. First, the current sample reflects a broader racial composition than our previous study. In addition, Dementyev et al. (12) was conducted during the 2020–2021 school year at the height of the COVID-19 pandemic, involving a limited cohort from 20 North American schools, most of which were under pandemic restrictions. In comparison, the current study was conducted post-peak COVID-19 during the 2021–2022 school year, included 31 schools, and had more than three times as many responses, with most schools open. Post COVID-19, many adolescents are facing a complex web of social and emotional challenges that are not captured in this study and cannot be expected to be resolved through participation in a cycling program alone. Indeed, the lack of significant change in relative clinical risk for mental health disorders following the R4F program is noteworthy. This stability suggests that the program may influence self-reported well-being and symptom awareness. However, on its own, the R4F program may not substantially alter the underlying risk for clinical mental health disorders in the short term. These findings align with research indicating that mental health interventions often require sustained effort and time to produce clinically significant changes (101–103). Overall, this study provides insights on how numerous factors contribute to the mental health and well-being of a broader sample of American adolescents and explores recovery following pandemic-era restrictions.

### 4.1 Gender

The current study builds upon our previous research demonstrating that participating in the R4F program is associated with better psychosocial well-being in adolescents, as evidenced by increased WHO-5 scores (12). Our findings regarding gender differences in mental health outcomes align with both our earlier work and the broader literature (29, 95, 104). Consistent with previous studies, our results show that males generally report better psychosocial well-being and a reduced risk of potential mental health issues compared to females (12). This gender disparity in mental health outcomes has been well-documented, particularly in the context of the COVID-19 pandemic (2, 104). Research suggests that females report lower mental well-being and are at greater risk for poor mental health outcomes compared to males (105–109). These differences have been attributed to a combination of biological and non-biological factors. These include gender-specific stressors, societal expectations, differences in mood symptom development, disproportionate hormonal transitions, lower life satisfaction, and higher levels of psychological distress among females (2). Additionally, females tend to participate in PA less frequently compared to their male counterparts (110), which may also contribute to these disparities in mental health outcomes (95, 104). However, it's important to note that the relationship between PA and mental health is complex and understudied, with mental health status potentially influencing PA habits and vice versa (111–113). Promoting PA opportunities that are supportive and tailored to the interests of female students is essential for beginning to close the gender gap in participation (110). Epidemiological evidence consistently demonstrates a pronounced female predominance in the prevalence of depression, with women being approximately twice as likely as men to experience depressive symptoms. Although studies suggest a narrowing of this gender disparity, the divergence typically emerges around age 12, peaks during mid-adolescence (around age 16), and subsequently declines, stabilizing in adulthood (105, 106, 108, 109). Our findings regarding the potential prevalence of psychiatric disorders across genders align with the existing literature, which consistently reports higher rates of mood, anxiety, trauma-related, and depressive disorders in females. Our findings regarding the prevalence of specific types of disorders among genders are also consistent with existing literature such as increased mood, anxiety, trauma-related, and depressive disorders seen in females and (105, 106, 108, 109).

### 4.2 Race

Studies often show that adolescents from racial and ethnic minority backgrounds report higher rates of experiencing mental and behavioral health (MBH) conditions-including anxiety, depression, ADHD, impulse control disorder, but are also less likely to access treatment services (114). This lack of care often results in more persistent and more severe MBH conditions. Additionally, racial and ethnic minority adolescents are less frequently offered high-quality, evidence-based care and are more likely to face adverse stressors early in life, such as toxic environments, cultural hardships, and macro-societal factors (115, 116). However, in contrast to this previous work, we did not observe consistent differences in mental well-being outcomes between students from underrepresented groups and white students. This is in line with the most recent Youth Risk Behavior Surveillance System (YRBSS) data from the CDC (88), whereby across most mental health measures, there were not significant differences across racial and ethnic groups. Of course, factors contributing to overall mental health are complex, and several factors may have contributed to this change. For example, our earlier study was conducted during the COVID-19 pandemic—a period that disproportionately affected the mental health of UG students compared to their White counterparts (84, 117). In addition, these results highlight potential differences among the schools that chose to implement the R4F program compared to those that do not, as they are offering increased access to additional programming to their students. Such opportunities, including tailored social-emotional learning curricula, targeted teacher and student training programs, and increased access to after-school programming opportunities are all associated with better mental health outcomes among students (118–121). Ultimately, identifying the precise and complex factors underlying these findings is beyond the scope of our current study, but warrants additional investigation given the potential mental health benefits for students.

### 4.3 Socioeconomic status

We did not observe any significant differences in student well-being across students from low and high SES backgrounds. This result is surprising based on previous research on socioeconomic disparities in adolescent mental health, which typically shows a higher prevalence of externalizing, internalizing, and attention behavior issues among students from low SES households (122–124). Two possible reasons for the lack of difference may be due to the schools that tend to implement the R4F program, as well as reliance on student self-report data. Many students either were not aware of their free or reduced lunch status (39% stated “I don’t know”) or did not want to share it, which could lead to student self-report of free and reduced lunch being less reliable. Secondarily, the wide range of social vulnerability indices among the sampled schools reflects the diversity in socioeconomic context among the sampled participants, which may contribute to the differences in student well-being that could impact study outcomes.

However, students from low SES backgrounds reported higher levels of well-being (WHO-5) after participating in the R4F program compared to before the program. Yet, there was not a statistically significant change in students from high SES backgrounds. These findings contrast with our previous work (12). This difference may reflect the changing influence of COVID-19-related stressors on low SES student mental health, which may have been more prominent in our earlier cohort but less so in this more recent, post-pandemic lockdown group (125).

Lower SES households are likely to have reduced access to sport facilities, related equipment, and other activities known to improve mental health (113, 126), which may have been exacerbated by the pandemic (127, 128). The subsequent school year may have seen a restoration of these opportunities, contributing to the observed improvements (125). This underscores the potential importance of easily accessible, school based, programs for adolescents from low SES backgrounds, such as the R4F program. Schools may be uniquely positioned to intervene in the traditionally bidirectional, positively-reinforcing relationship between low SES and poor mental health by providing such programs (129–131). However, these differences could also be attributed to factors not accounted for in this study, such as geographical and economic barriers, nutritional status, or other variables that interact with socioeconomic status to influence mental health outcomes. Further research is needed to elucidate these potential confounding factors and their impact on the effectiveness of programs like R4F across different socioeconomic groups.

### 4.4 Club involvement

Students involved in extracurricular clubs exhibited higher levels of psychosocial well-being both before and after participating in the R4F program. Numerous studies support this observation, demonstrating that participation in extracurricular activities predicts positive outcomes among adolescents including improved social belonging, reduced depression, better mental health, increased civic engagement, and higher educational achievement (132–135).

However, participating in the R4F program was associated with better WHO-5 scores for students who were not engaged in clubs. This suggests that structured PA programs offered during the school day in a non-competitive setting can provide positive avenues for social interaction, confidence-building, and improved well-being. This finding aligns with research highlighting the psychological, educational, and social health benefits of participation in club-based or team-based sports (103, 136, 137). While the R4F program may not fully replace the positive aspects of extracurricular club involvement, it appears to offer valuable opportunities for enhancing mental health and well-being, particularly for students with limited access to traditional extracurricular activities.

Examining the common elements between clubs and the R4F program could provide valuable insights for shaping school policies. Both clubs and the R4F program offer opportunities for skill development, social interaction, and a sense of belonging, which are crucial factors in promoting adolescent well-being. The quality of experiences in organized activities, rather than mere participation, may be key to understanding their developmental benefits (138). Integrating structured PA programs like R4F into the school day could complement existing extracurricular offerings and provide more equitable access to the mental health benefits associated with organized activities. Future research should focus on identifying the specific mechanisms through which these programs enhance adolescent well-being and how they can be optimally implemented in diverse school settings.

### 4.5 Breakfast

Participation in the R4F program was associated with increased WHO-5 scores, regardless of whether students ate breakfast daily or not. However, students who ate breakfast daily had significantly higher baseline and post-program WHO-5 scores than those who did not eat breakfast daily. This aligns with existing evidence suggesting that breakfast consumption is associated with improved mental health and well-being in adolescents (79, 139, 140).

In addition, we observed a dose-dependent relationship between the number of days students reported eating breakfast and their psychosocial well-being, a finding supported by current literature (79, 141). For instance, a nearly linear relationship has been observed between higher frequency of breakfast consumption and greater life satisfaction in children and adolescents (79). To build on these findings, future research should explore not only the frequency of breakfast consumption but also the quality of the breakfast consumed in relation to PA, given their potentially synergistic impact on student psychosocial well-being and associations with better mental health outcomes in adolescence (139, 141). Indeed, as cycling can be a vigorous activity, ensuring students are properly fueled before going for a bicycle ride can ultimately impact their experience with the activity.

### 4.6 Physical activity

We observed higher WHO-5 scores following participating in the R4F program compared to before participating in the program, regardless of whether the students were regularly active outside of the program. However, both at baseline and after participating in the program, students who were active at least 4 days a week had higher WHO-5 and lower PSC-17-Y scores, aligning with previous work (12, 142). In addition, students who reported being active at least 4 days a week also had lower externalization, internalization, and attention sub scores both pre- and post-program compared to those who were not regularly active. This finding is consistent with existing literature (143, 144), which suggests that PA can be an important tool that helps reduce anxiety, mood disorders, and ADHD symptoms.

We also saw a clear dose-dependent relationship between the number of days of PA reported and the WHO-5 and PSC-17-Y scores. In line with current PA recommendations (27, 88, 95), we observed the highest levels of student well-being, across both measures, in those who reported being active 7 days a week. However, this must be balanced against the observation that those already meeting PA recommendations experienced only modest increases in their mental health and wellness scores. These findings illustrate that further research is needed to resolve the optimal frequency and intensity of PA for maximizing mental health benefits in adolescents (95, 104).

### 4.7 Screen time

The relationship between PA, screen time, and adolescent mental health is complex and increasingly relevant in our digital era. Our study builds upon previous research and provides new insights into this relationship, particularly regarding the impact of interventions like the R4F program (12). Consistent with existing literature, we found that students adhering to screen time recommendations generally reported better psychosocial well-being (98, 145, 146). In addition, students who exceeded the recommended screen time of 2 h per day also reported significantly higher PSC-17-Y externalization, internalization, and attention sub scores than students who met screen time recommendations. This observation is consistent with studies reporting associations between excessive screen time use and various mental health challenges, including behavioral issues, emotional problems, and attention difficulties (147, 148). However, we also observed that participating in the R4F program was associated with larger changes in mental well-being for students who did not meet screen time recommendations compared to those who met the recommendations. While more research is needed, cycling may be a particularly promising intervention for adolescents with higher screen time use, an important consideration given the increasing time young people spend on electronic devices. However, the persistence of the gap in WHO-5 scores, PSC-17-Y composite scores, and PSC-17-Y sub scores between the two groups indicates that the benefits of limited screen time remain significant even after the intervention.

Our study also identified a dose-response relationship between screen time and mental health outcomes. The negative association between screen time use and WHO-5 scores, coupled with the positive association with both the PSC-17-Y composite and sub scores, suggests that even incremental increases in screen time may have negative impacts on mental health. This relationship persisted after the R4F program, indicating that the impact of screen time on mental health is robust and cannot be fully offset by PA intervention on its own (145, 149–151). Further, while PA interventions like R4F show promise in improving some mental health outcomes (12, 145, 149), they do not comprehensively address all aspects of psychosocial well-being, especially for heavy screen users. The program's limited impact on certain mental health risks associated with excessive screen time warrants further investigation.

### 4.8 Sleep

The influence of sleep on adolescent psychosocial well-being and the efficacy of the R4F program is a critical area of investigation, given the importance of adequate sleep for mental health (12, 99, 100). Our findings provide compelling evidence for the significant role of sleep in adolescent mental health and its interaction with PA interventions. Both before and after participating in the R4F program, students meeting sleep recommendations demonstrated markedly better psychosocial well-being than those who did not. Additionally, students who slept at least 8 h a night exhibited lower baseline externalization, internalization, and attention sub scores. These results align with existing literature emphasizing the positive impact of adequate sleep on various aspects of mental health and cognitive function in adolescents (152, 153).

Participation in the R4F program was associated with modest improvements in student well-being, regardless of meeting sleep recommendations, consistent with our previous findings (12). However, sleep is an important factor contributing to overall mental well-being, and participating in R4F alone is not enough to overcome poor sleep. Indeed, large gaps in well-being scores persisted between those who did and did not report meeting sleep recommendations.

Our analysis of relative risk for developing psychosocial illness reveals a concerning trend and again supports our earlier work (12). Students not meeting sleep recommendations consistently demonstrated higher rates of poor mental health and an elevated risk for psychosocial disorders, both before and after the R4F program. This finding highlights the critical role of sleep in mental health resilience and suggests that PA interventions alone may not be sufficient to mitigate the risks associated with inadequate sleep.

The observed dose-response relationships between sleep duration and mental health outcomes provide valuable insights into adolescent well-being. Our findings reveal a positive association between sleep duration and WHO-5 scores and a negative association between sleep duration and PSC-17-Y composite and sub scores. Each additional hour of sleep incrementally improved mental health and well-being scores in the adolescents of our study. These results align with current literature (150), which emphasizes the importance of adequate sleep for mental health. Importantly, even modest increases in sleep duration were associated with measurable improvements in mental health outcomes. These findings underscore the independent and enduring role of sleep in adolescent mental health, highlighting the need for sleep-focused interventions alongside PA programs to comprehensively support adolescent well-being.

## 5 Limitations

Our investigation into the R4F program's effects on adolescent mental health and well-being in a post-pandemic cohort reveals a nuanced landscape of outcomes. The results, while partially consistent with our earlier findings, also demonstrate some departures, underscoring the intricate nature of mental health interventions for adolescents (12). While the anonymous nature of our surveys, the lack of a comparison group, and the pandemic-emergent context influenced our ability to draw causal conclusions, the study nonetheless provides valuable insights into the program's potential impact, and the relationships between important lifestyle factors and overall well-being in adolescents. Although we observed many meaningful relationships between lifestyle factors and well-being outcomes, the absence of a control group limits our ability to attribute changes in WHO-5 and PSC-17-Y scores to the program itself rather than to other developmental, social, or environmental influences adolescents may be experiencing. This limitation persists despite additional qualitative data, which we are currently analyzing for potential efficacy-related insights suggesting program effectiveness. The lack of congruence between WHO-5 and PSC-17-Y outcomes observed across the various subgroups following the R4F program may also reflect the differential sensitivity of each instrument to mood, behavioral, and attentional domains (51, 53, 63, 66, 68). This could also arise because the PSC-17-Y has not been fully validated, and thus it is unclear if the observed score changes reflect true shifts in symptoms or are within the range of expected measurement variation. This divergence between the two assessments could signal selective program benefits or increased student awareness of psychosocial symptoms, effects which are possibly enhanced by engagement with the R4F program. Such patterns highlight the complexity of adolescent mental health and the necessity of multimodal, longitudinal, and context-sensitive assessment strategies in future research.

To strengthen future research, comparison groups must be included, and longitudinal tracking of individual students is recommended. Future research may also consider incorporating physiological or behavioral measurements such as heart rate monitoring, accelerometry, cardiorespiratory fitness, or sleep assessments to better understand the activity levels and student responses to the program (154–157). These enhancements will provide a more robust evaluation of how cycling programs influence adolescent mental health, allowing us to better understand and optimize their effectiveness in supporting student well-being.

We opted to prioritize a more exploratory approach in this preliminary phase of the study due to its simplicity and interpretability whereas future studies would benefit from statistical sophistication. In this case, single-level analyses were easier to conduct, interpret, and communicate findings for non-technical stakeholders. While preliminary results from our study yield promising information, these results should be interpreted with caution as a result of the study analysis. Our current approach sought to treat participant observations independently, which could potentially underestimate standard errors and produce overly narrow confidence intervals, likely increasing a Type I error rate. Given the hierarchical nature of our data, students nested within schools, future analysis would benefit from a multilevel modeling approach to provide effect estimates of school-level variation in program effectiveness. Further, our use of listwise deletion for missing data may have introduced bias, whereas a multilevel modeling approach can handle missing data through techniques like maximum likelihood estimation.

## 6 Perspectives

This study underscores the importance of in-school cycling programs and their potential to impact adolescents' mental health. Building on our previous pandemic-era research, our work demonstrates a modest association between participation in the program and overall mental health and well-being among participants in the COVID-emergent R4F program. These findings highlight the potential benefits of in-school cycling initiatives for enhancing the mental health and wellness of middle school students in the United States. Beyond the R4F program, our research highlighted how various factors, including race, gender, socioeconomic status, club involvement, breakfast consumption, PA levels, screen time, and sleep duration, can also play a role in understanding student well-being. These intricate relationships emphasize the need for tailored interventions that address the diverse needs of student populations, as well as further research to better understand and effectively target these multifaceted influences on adolescent mental health. By acknowledging these complexities, we can work towards developing more comprehensive and effective strategies to support the mental well-being of middle school students through cycling programs and other targeted interventions.

## Data Availability

The raw data supporting the conclusions of this article will be made available by the authors, without undue reservation.
